# Review on Geopolymers as Wellbore Sealants: State of the Art Optimization for CO_2_ Exposure and Perspectives

**DOI:** 10.1021/acsomega.3c01777

**Published:** 2023-06-23

**Authors:** Seyed Hasan Hajiabadi, Mahmoud Khalifeh, Reinier van Noort, Paulo Henrique Silva Santos Moreira

**Affiliations:** † Department of Energy and Petroleum Engineering, Faculty of Science and Technology, University of Stavanger, 4036 Stavanger, Norway; ‡ Department of Reservoir Technology, Institute for Energy Technology, Postbox 40, 2027 Kjeller, Norway

## Abstract

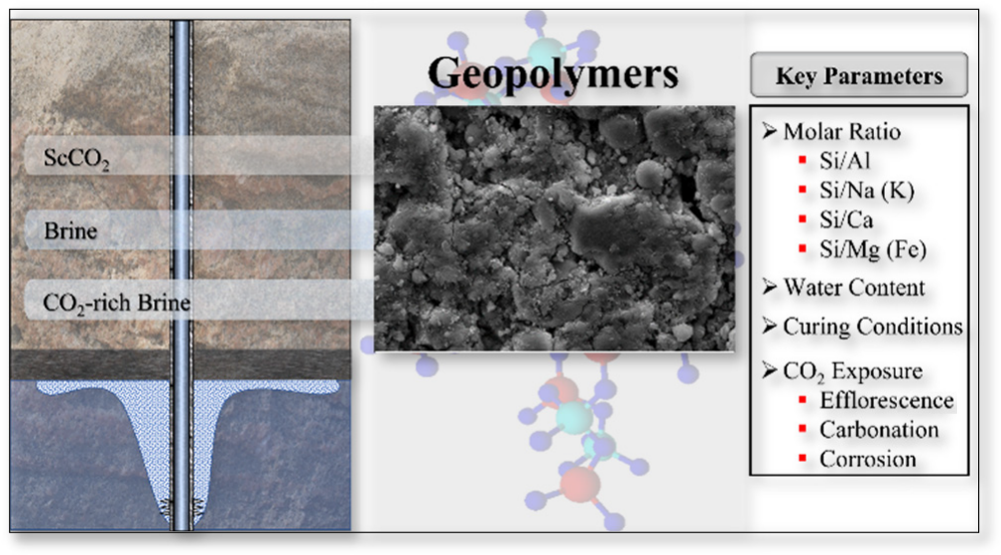

Wellbores used in underground production and storage activities, including carbon capture and storage (CCS), are typically sealed using sealants based on Ordinary Portland Cement (OPC). However, leakage along these seals or through them during CCS operations can pose a significant threat to long-term storage integrity. In this review paper, we explore the potential of geopolymer (GP) systems as alternative sealants in wells exposed to CO_2_ during CCS. First, we discuss how key parameters control the mechanical properties, permeability, and chemical durability of GPs based on different starting materials as well as their optimum values. These parameters include the chemical and mineralogical composition, particle size, and particle shape of the precursor materials; the composition of the hardener; the chemistry of the full system (particularly the Si/Al, Si/(Na+K), Si/Ca, Si/Mg, and Si/Fe ratios); the water content of the mix; and the conditions under which curing occurs. Next, we review existing knowledge on the use of GPs as wellbore sealants to identify key knowledge gaps and challenges and the research needed to address these challenges. Our review shows the great potential of GPs as alternative wellbore sealant materials in CCS (as well as other applications) due to their high corrosion durability, low matrix permeability, and good mechanical properties. However, important challenges are identified that require further research, such as mix optimization, taking into account curing and exposure conditions and available starting materials; the development of optimalization workflows, along with building larger data sets on how the identified parameters affect GP properties, can streamline this optimization for future applications.

## 1 Introduction

Carbon capture and storage (CCS), with storage in porous geological reservoirs of sedimentary origin, is considered a key technology for the mitigation of climate change.^1−4^ An important challenge for geological CO_2_ storage is the integrity of the wells used during CO_2_-injection (as well as potential pre-existing wells). The main risk to the sealing integrity of these wells is leakage through the cement matrix or along the cement-formation and cement-steel interfaces, which could lead to the uncontrolled release of the injected CO_2_.^5−7^ In most CCS operations, CO_2_ is injected to a depth of 800 m or greater, where required conditions for the supercritical state of CO_2_ (scCO_2_) are met (i.e., pressures greater than 7.38 MPa and temperatures above 31.04 °C). A typical geothermal gradient in such a deep storage reservoir is in a range of 20–40 °C/km, and brine salinity ranges between 0 and 40%. Furthermore, when CO_2_ is injected into underpressured depleted fields, the thermal stresses due to the Joule–Thompson effect can add more complexity to the required attributes of wellbore isolation systems.^8−11^ The aggressive environment created can result in degradation of Ordinary Portland Cement (OPC) in the presence of CO_2_ or low pH CO_2_-saturated brine, potentially leading to increased permeability and mechanical failure due to changes in effective stress and (cyclical) temperature variations.^12−14^ For instance, Nelson^15^ noted a significant increase in water permeability of specimens made of class G cement after a one-month period of aging in CO_2_-rich environment, reaching values of around 10 to 100 times greater than the recommended limits. Similar results are reported by other researchers for OPC exposed to wet or dry CO_2_.^16−18^

These challenges have encouraged researchers to seek alternative materials, such as calcium sulfoaluminate/aluminate cement, supersulfated cement, pozzolanic-based slurries, thermosetting resins, unconsolidated materials, bismuth-based metals, alkali-activated materials (AAMs), and geopolymers (GPs).^19−22^ GPs are a subcategory of AAMs, characterized by long-chain polymeric bonds (e.g., Si–O–Al–O) and low Ca content. They can be produced by mixing a liquid activator (i.e., hardener), such as solutions of K_2_SiO_3_, Na_2_SiO_3_, NaOH, KOH, Na_2_CO_3_, and K_2_CO_3_, with various sources of (solid) reactive aluminosilicate precursors. The precursors most considered are fly ash, metakaolin, blast furnace slag, naturally occurring rocks, natural soil, biomass ash, rice husk ash, red mud, mine tailings, and glass product waste.^23−28^ Some of the major benefits of GPs over OPC-based binders are their environmental and economic merits, low Ca content, simple synthesis process, lower chemical shrinkage, rapid strength development, low matrix permeability, resistance to fire and acid attack, and the potential to immobilize toxic materials.^2,29−36^ However, these properties can vary significantly depending on the mix design of the slurry and the minerology of the GPs.

Although the modern development of AAMs goes back to 1959, the study of GPs as green alternatives to OPC started coming into focus approximately 40 years ago.^37−41^ Since then, the notable features of GPs have attracted the attention of academic and industrial sectors, resulting in increasing research intensity as well as progressively extensive application of GPs, such as in concrete, coating applications, refractory materials, insulation, and fire-resistant materials.^42,43^ GPs have also been recognized as suitable candidates for zonal isolation of CCS wellbores mainly because of their lower permeability and lower Ca content compared to OPC-based sealants.^32,44−46^ However, serious challenges to the use of GPs in zonal isolation of CCS wells do exist and need to be dealt with. Key issues noted in the literature include the sensitivity of GP activation and polymerization to water content, the viscosity profile, controlling thickening time, efflorescence (alkali leaching) and/or carbonation, and the low tensile strength of GPs.^46−49^ Moreover, due to the system complexity caused by the wide range of parameters involved in GP synthesis, development of standard mix designs is still in its primary stage (laboratory scale). The need for liquid alkali-based activators with high pH imposes further challenges due to safety issues, and studies working on reducing the need for such activators are broadly welcomed.^23,50^ Finally, while GPs have been recognized as potential wellbore sealant materials, and despite the extensive research conducted on the development of GP systems and a noticeable number of reviews published on various aspects of these materials,^28,29,42,51−61^ the zonal isolation potential of GP systems in CCS operations has not been addressed as thoroughly.

The present work aims to identify and fill the gaps by discussing the major parameters contributing to the sealant behavior of a GP system exposed to the physically and chemically aggressive environment of a CO_2_ injection wellbore and by exploring the optimized value of each parameter, leading to the best performance of the GP. Through this review, shortcomings and knowledge gaps requiring further research and development have been identified and discussed.

## 2 GP Systems

As stated above, GPs are alkali-activated materials (AAMs) typified by low Ca content and the formation of long-chain polymeric bonds. The reaction steps involved in the hardening process are (a) dissolution/depolymerization, (b) transportation, (c) nucleation and coagulation, and (d) polycondensation/geopolymerization.^62^

For GPs, as low Ca-content materials, the alkali excitation occurring throughout the first reaction step breaks chemical bonds within the aluminosilicate precursor minerals and decomposes these structures into a silicon–oxygen tetrahedron and an aluminum–oxygen tetrahedron, in which the basic monomeric form is generally represented as [(OH)_3_–Si–O]^−^ and [(OH)_3_–Al–O]^2–^. Subsequent interactions between these monomers leads to the formation of dimers and then trimers, tetramers, oligomers, etc.^63,64^ Further rearrangement and polycondensation of these structures lead to the creation of a three-dimensional network structure of semicrystalline aluminosilicate particles. Noteworthy is that sodium aluminosilicate hydrates (N–A–S–H) or potassium aluminosilicate hydrates (K–A–S–H) are denoted as GP gels if they are developed but not yet fully condensed.^20,51,62,65,66^

In high Ca-content aluminosilicate materials, calcium silicate hydrate “C–S–H” (or calcium aluminate silicate hydrate “C–A–S–H” when significant Si^4+^ is replaced by Al^3+^) will also form.^67−69^ As C(−A)–S–H gels are also among the reaction products of OPC hydration, the behavior of these gels is very well described in the literature. However, major uncertainties remain with regard to the properties and behavior of (N,K)–A–S–H gels.^70−73^

### 2.1 Precursors in a GP System

Comprehensive descriptions of precursor materials used in GP systems are presented in the works of Provis and Van Deventer^63^ and Freire et al.^46^ This section presents a brief overview of the key precursor materials, focusing on how they impact GP properties that are of importance for CCS applications.

#### 2.1.1 Fly Ash and Bottom Ash

**Figure 1:**
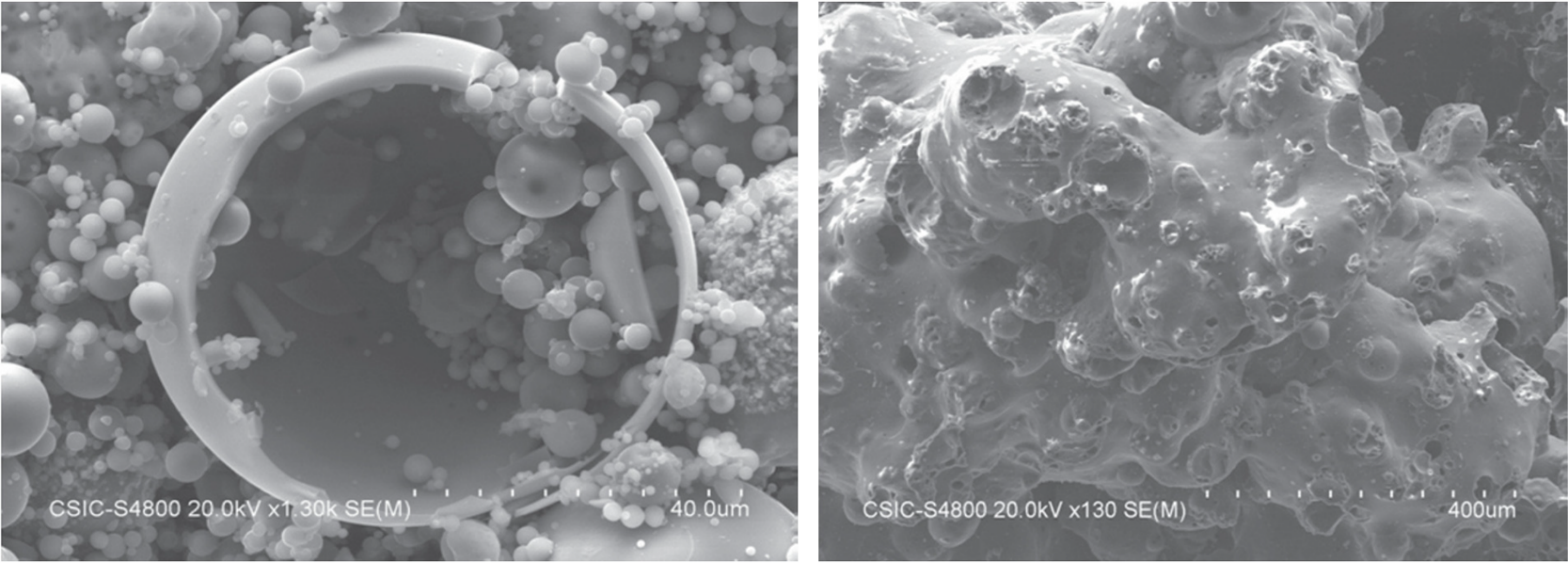
SEM images representing fly ash (left) and bottom ash (right). Reproduced with permission from ref (79). Copyright 2014, Elsevier.

Fly ashes are aluminosilicate materials, consisting of finely sized spherical particles, with an average composition of 40–60% of SiO_2_ and 20–30% of AlO_4_.^55,74,75^ While in ASTM C618–03, fly ashes were classified into low Ca-content (Class F, less than 10 wt % Ca) and high Ca-content (Class C, more than 10 wt % Ca) materials, and in the most recent ASTM C618–19, this limit has changed to 18 wt % Ca content.^76^ Fly ashes are among the most studied GP precursors, due to their low price, availability, mineralogical suitability, and the high mechanical durability of the resultant GPs.^28,32,77,78^ Bottom ash is another well-known ash but consists of angular porous particles, with a rough surface texture (see Figure 1).^79^ Compared to fly ash, the use of bottom ash in GP production is much more limited, mainly due to its coarser particle size, lower reactivity, and consequently the lower compressive strength of the synthesized GPs. However, more recently, GPs with promising properties have been produced from precursors containing both fly ash and bottom ash.^57,80−83^

The strength development of fly-ash-based GPs requires relatively high curing temperatures (between 55 and 90 °C); however, this can be adjusted by grinding of the fly ash or addition of various amounts of slag, gypsum, calcium aluminate cement, or OPC to the precursor mixture.^28,44,65,84−86^ In general, fly-ash-based GPs show higher compressive strength, lower density, lower shrinkage, and lower Young’s modulus (i.e., greater ductility) compared to OPC.^23,87^ On the other hand, it is reported that AAMs synthesized with high Ca-content fly ashes suffer from significant swelling issues in the presence of water as well as notable drying shrinkage under atmospheric conditions.^84^ It is also worthy to note that, due to the great chemical and physical heterogeneity of fly ash particles, even within a single particle GPs prepared from various sources of fly ash can exhibit significantly different characteristics.^51,88^ Therefore, normalization of elements present in the precursors, by the addition of another precursor material, may be required to create standard chemical compositions.

#### 2.1.2 Metakaolin

**Figure 2:**
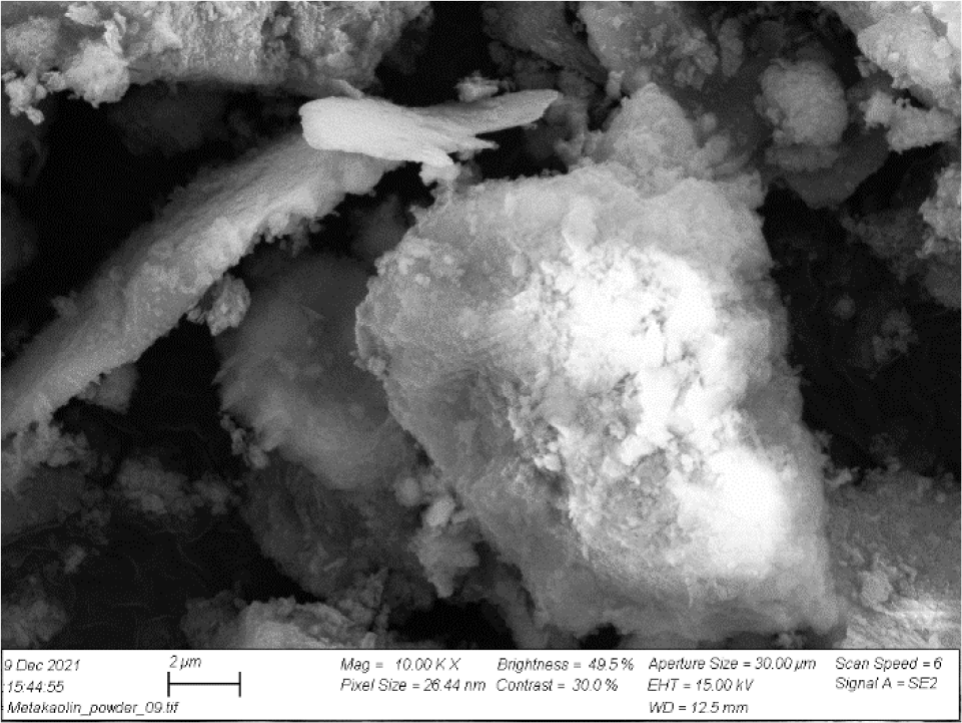
SEM image representing the plate-like shape of metakaolin particles.

Another widely applied precursor material is metakaolin. This is produced by the calcination of kaolinite, a clay mineral, at temperatures between 500 and 800 °C.^63^ Compared to GPs based on uncalcined kaolinite, metakaolin-based systems benefit from higher precursor reactivity (due to its amorphicity), leading to faster rates of dissolution/gelation and higher compressive strengths.^31,46,89^ Compared to other GP precursors, metakaolin-based GPs also take advantage of the simplest alkali activation process required for GP synthesis, and their three-point flexural strengths are close to those of OPC (5–6 MPa).^63,90^ However, in general, metakaolin-based GPs show lower durability, mainly caused by the plate-like shape (and thus higher surface area) of metakaolin particles, the resulting higher water demand, and, consequently, higher risks of drying shrinkage and cracking (Figure 2).^31,63,91^ Furthermore, the plate-like shape of metakaolin particles results in delayed setting and strength development, which can in turn lead to the evaporation of the hardener phase prior to the occurrence of full polycondensation. Even if the specimen is sealed, when a sample is cured at a high temperature, removing the sealing material can cause sudden liquid evaporation, leading to elevated pore pressures in excess of the mechanical strength of the specimen. Accordingly, fractures and cracks will appear, especially at high curing temperatures applied throughout an extended period of time.^19,67,89,92−94^

#### 2.1.3 Rock-Based Precursors

Precursors based on rocks such as granite, aplite, and norite have recently attracted considerable attention, mainly because of the abundance and availability of these materials.^70,92,95−97^ Of late, Davidovits,^98^ credited with the invention of the term “geopolymer”, emphasized the application of rock-based GPs as the main pathway for reducing the production of CO_2_ associated with OPC-based concretes. When used as a GP precursor, the selected rock material will be milled, to increase particle reactivity, and it may undergo additional mechanical or thermal activation to further enhance reactivity, though for these materials thermal activation does not necessarily result in the formation of amorphous phases such as formed during kaolinite calcination.^92,99,100^

Granite is a felsic plutonic rock with high silicon and aluminum content and medium to coarse grains, and it is among the most common plutonic rocks in the upper continental crust.^27,101,102^ Granite is a promising candidate for GP synthesis because of its chemical composition and its prevalence worldwide.^30,103,104^ Mineralogically, granite mainly consists of quartz, plagioclase, K-feldspar, and biotite.^101,102^ Granite-based GPs can be synthesized through alkali activation or alkali fusion processes. Alkali fusion includes alkali thermal preactivation of less reactive precursors by calcination of a dry mixture of these raw precursors with alkali hydroxides, at temperatures above the alkali hydroxide melting point. The resulting fused materials are then ground and activated by using an alkali silicate activator. It should be noted that, during this step, geopolymerization does not take place via dissolution but rather through transportation and polycondensation steps.^30,105,106^ Laboratory assessments of granite as GP precursors show that they provide high reactivities, sufficient strength development, and workable setting times.^107^

Aplite is a Na-rich, granitic rock with high concentrations of SiO_2_ and Al_2_O_3_, which mainly contains alkali-feldspars, quartz, muscovite, oligoclase, and, more rarely, biotite. However, compared to other granites, aplite shows higher reactivity due to its inherently finer crystal size (under 1 mm across).^92,108^ Norite, a medium-to-coarse-grained mafic plutonic rock consisting mostly of plagioclase and orthopyroxene, is another rock-based precursor that has shown desirable results in laboratory studies.^101^ This rock is particularly suited for use as a GP precursor due to its high aluminosilicate (plagioclase) content.^70,101^

While rock-based precursors exhibit relatively low reactivity during the early phase of polymerization and have relatively high Si/Al-ratios, these properties can be improved through the addition of reactive amorphous ingredients such as slag, fly ash, microsilica, or metakaolin.^67,70,109^ As rock-based precursors have not been studied as widely as other precursor materials, various uncertainties remain, especially regarding the emplacement and scalability of such GPs. Further research and development are thus required to enable large-scale application of rock-based GPs.

#### 2.1.4 Waste Glass

Due to its high silica content and amorphous nature, waste glass, especially when supplemented with alumina-rich materials such as fly ash, metakaolin, and calcium aluminate cement, can be used as a precursor for GP production. While some studies suggest that glass-based GPs could even be composed without additional alumina, improved durability and higher compressive strengths reported for GPs synthesized from a combination of glass and alumina-rich materials do highlight the vital role of alumina content in GP strength development and durability.^110−112^ Noteworthy is that recent studies exhibited that waste glasses, especially when treated with solutions of NaOH/Na_2_CO_3_, can act as proper alkaline activators which can induce the same impacts as silicon in water glasses.^113−115^

### 2.2 Additives

While it is also occasionally considered as a precursor, ground granulated blast furnace slag (GGBFS) is more frequently used as an additive (i.e., minor component), to adjust the chemical composition by introducing a significant amount of calcium, and to increase the reactivity by adding amorphous content; to modify setting time, durability, and strength development.^116−119^ The composition of GGBFS varies, depending on the specific ores and furnaces; however, it mainly consists of poorly crystalline phases and some depolymerized calcium silicate glasses.^63^

As a raw material with high Ca content, GGBFS mostly increases the reactivity of a GP precursor mix as well as the rate of precipitation of C(−A)–S–H gels.^85^ Experimental studies have confirmed the coexistence of C(−A)–S–H and N–A–S–H gels in slag-based systems.^63,67,68,70,109,120^ In this way, the use of slags in GP systems leads to internal confinement and increased compressive strengths, at least through early stages of geopolymerization.^121,122^ However, the addition of GGBFS to precursors may increase the likelihood of cracking and reduce the durability of the resultant GP.^19^

GP properties can also be enhanced effectively through the use of microsilica, also known as silica fume. Microsilica mainly consists of amorphous particles of SiO_2_ (in the form of ultrafine spheres) that act as highly pozzolanic, reactive microfillers, reducing permeability, enhancing corrosion resistance, and improving durability.^92^ The use of this additive, due to its extremely high fineness and the resulting increase in the Si/Al ratio of the system, can also result in improved compressive strength, abrasion resistance, and GP-to-casing bond strength.^19,123,124^ Microsilica is also sometimes reported to increase geopolymerization, by supplying additional nucleation sites. On the other hand, excess concentrations of microsilica have been linked to reduced durability due to excessive self-desiccation and cracking.^123,125^

### 2.3 Hardeners in a GP System

Hardeners, also known as alkali reactants, mainly include alkali hydroxides and alkali silicates. However, the use of alkali carbonates and alkali aluminates has also been reported.^51^ The major roles of alkali reactants are (a) to provide high pH conditions required for the activation of the raw aluminosilicate precursors and (b) to provide alkali cations that can balance the negative charges induced when AlO_4_ tetrahedra are incorporated into the cross-linked 3D network structure of the GPs.^7,35,46,57,126,127^

The main properties of the hardeners affecting the resultant GPs are the concentration and composition of the alkali solution. Through controlling the reaction rate, alkali content has a strong impact on the development of GPs’ mechanical properties.^48^ When the concentration of alkali cations is too low, this can lead to a lower reaction rate mainly because of the incomplete dissolution of raw materials. In contrast, excess alkali contents result in reduced ionic activity due to electrostatic shielding and can hinder the precipitation of new solid phases.^26,43,128^ In addition, excess alkali content leads to an increased risk of alkali leaching-related issues (see Section 3.4.1).^63^ Therefore, it is important to determine the optimum concentration of alkalis for each GP synthesis process, to ensure a high degree of geopolymerization while limiting electrostatic shielding of ions.^26,31^ In this regard, considerable variation in the optimum alkali concentration has been reported in the literature. For instance, Panagiotopoulou et al.^129^ found an optimum concentration of 10 M for NaOH, while the optimum molarity reported by Zhang et al.^31^ and Panias et al.^87^ was 15 and 6.6 M, respectively. For fly-ash-based GPs, Görhan and Kürklü^130^ and Cao et al.^65^ found an optimum concentration of 6 and 12 M for NaOH, respectively, while Somna et al.^131^ observed a sharp increase in the mechanical strength of GPs at NaOH concentrations up to 9.5 M, followed by a moderate trend at NaOH molarities between 9.5 and 14 M and a decreasing trend at molarities higher than that. These variations likely derive from the different degrees of dissolution of alumina and silica, the type and composition of source materials used in each study, different curing conditions, and differences in the pozzolanic reactions forming C(−A)–S–H gels.^31,51^

Considering the effects of alkali type, most studies show the greater efficiency and higher reactivity of Na-based activators compared to K-based species, explained by the smaller ionic radius of Na^+^ (around 116 pm) compared to that of K^+^ (around 152 pm), enhanced ionic pair reaction with smaller silica oligomers, and easier mobilization of ions through the gel network. However, others^51,130−136^ report that the greater ionic radius of K^+^ can lead to lower surface charge densities and higher degrees of geopolymerization in the synthesized GPs.^86^ Furthermore, some experimental studies have shown K-based activators to result in lower slurry viscosities.^20,119^

The activation of precursors in the presence of insufficient soluble silicates (i.e., waterglasses) may lead to the formation of hydroxysodalite, rather than GPs, where the reduced formation of Si–O–Si bonds leads to a reduced degree of geopolymerization.^45,87^ Waterglasses included in the hardener enhance the rate of dissolution and polymerization and significantly affect the fresh and hardened attributes of GPs.^51,134,137^ Moreover, it is shown that GPs activated by a combination of waterglasses and alkali hydroxides benefit from enhanced mechanical strengths and microstructures due to the formation of gels richer in Si, smaller pore sizes, and the increased compactness of the material compared to GPs hardened with alkali hydroxides alone.^57,75,88,119,138,139^ However, excess quantities of waterglass can result in lower compressive strengths, impeded microstructure development, and chemical instability of GPs, especially in the presence of water, when the resulting formation of more Si-rich gels leads to a more amorphous GP structure, which in turn leads to leaching of unreacted silicates.^87^ For instance, Pavithra et al.^140^ observed an increasing trend of compressive strengths with increasing ratio of Na_2_SiO_3_/NaOH (up to 1.5), followed by a sharp decrease in compressive strength at higher Na_2_SiO_3_/NaOH ratios. Besides, increased molarity of NaOH up to 16 M resulted in higher compressive strengths, while higher molarities caused lower compressive strengths. In another study, Abdullah et al.^141^ obtained the highest compressive strength at an optimum molarity of 12 for NaOH, the Na_2_SiO_3_/NaOH ratio of 2.5, and a fly ash/alkaline activator ratio of 2. Furthermore, excess silica contents have been linked to reduced water evaporation,^28,42,79,142^ though experimental study has demonstrated increased water evaporation rates and increased pore pressure beyond the compressive strength.^92^ It is also worth noting that an increased ratio of waterglass to alkali hydroxide in solution can lead to increased slurry viscosities, thus negatively affecting the workability of the system.^81^ Limited experimental evidence shows better performance of hardener mixes based on a single alkali metal (NaOH/Na-silicate, KOH/K-silicate), leading to improved GP properties.^51^ Finally, it should also be noted that the differences between the performance of various activators (alkalis or waterglasses) might be due partly to the varying structure of alkaline silicates present in the hardener phase.^63,143^

## 3 Factors Affecting the Properties of GPs

Throughout the past decades, numerous attempts to optimize GP systems, using a wide variety of raw materials with varying chemical compositions, have been reported in the literature (see Table 1). A compilation of reported data shows that that the behavior of GP systems mainly depends on the chemistry of the precursor and hardener (i.e., element molar ratios such as Si/Al, Si/Ca, etc.), water to binder ratio (w/b), curing conditions, and environmental factors, including humidity and CO_2_ content in CCS applications.^20,68,128,144^ This section presents a brief review of these factors and their effects on the properties of GPs and a summary of the optimum values suggested in the literature.

**Table 1:** List of the Molar Ratios and Conditions of Some of the Precursors Used in the Literature

		Parameters						Curing conditions		
Authors	Precursor(s)	SiO_2_**/**Al_2_O_3_	SiO_2_/Na_2_O	SiO_2_**/**K_2_O	SiO_2_**/**CaO	SiO_2_**/**MgO	SiO_2_**/**Fe_2_O_3_	*w*/*b*	*T*	Other factors
Oderji et al.^145^	FFA^a^	2.20	85.91	39.21	7.22	51.73	10.10	0.28	Ambient	FFA/GGBFS = 0.85
	GGBFS^b^	2.85	128.7	135.63	0.84	5.99	83.50	0.30		
								0.32		
Assi et al.^124^	FA	1.86	66.88	N/A	33.44	66.88	7.13	0.28	Ambient (22 °C) and 75 °C	10% of FA was replaced by OPC to eliminate the external heat required
	OPC	4.38	N/A	N/A	0.31	14.07	5.63			
Khalifeh et al.^62^	Aplite rock	9.16	30.44	26.62	100.9	828	110.4	0.55–0.58	25–50 °C	-
	GGBFS	2.62	37.78	N/A	1.10	2.00	N/A			
Salehi et al.^144^	FFA	1.7–9.2	N/A	N/A	2.04–49.68	N/A	1.11–10	N/A	60–121 °C	-
Alvi et al.^146^	Granite normalized by silica flour and GGBFS	6.55	34.80	36.95	5.49	10.32	113.40	0.25	50 and 70 °C	Liquid to solid (L/S) ratio = 1.82
Khalifeh et al.,^19^ Khalifeh et al.,^92^ and Khalifeh et al.^67^	Aplite rock	9.16	30.44	26.62	100.9	828	110.40	4.55%	50 and 70 °C	Water content is based on percentage of total solid phase
	GGBFS	2.62	37.78	N/A	1.10	2.00	N/A			
	Micro silica	136.4	238.75	95.50	238.75	191	318.33			
Chamssine et al.^95^	Granite, normalized with other precursors having reactive properties	4.87	26.97	16.56	6.35	13.90	42.35	0.35	50 and 70 °C	-
Khalifeh^20^ and Khalifeh et al.^70^	GGBFS	2.62	37.78	N/A	1.10	2.00	N/A	N/A	23 and 87 °C	Different L/S ratios were considered (II; III; IV; V; VI; VII; VIII)
	Micro silica	136.4	238.7	95.50	238.75	191.00	318.3			
	Norite	2.77	12.65	47.78	6.52	6.32	3.44			
Wang et al.^128^	As-received lithium slag	2.45	N/A	138.74	10.03	N/A	42.85	0.65	25 °C	-
	Calcined lithium slag	2.39	N/A	123.96	10.98	N/A	35.42			
Kong and Sanjayan^147^	FFA	1.81	131.89	57.41	7.87	34.86	4.78	N/A	Ambient temperature to 800 °C	L/S ratio = 0.33
Hardjito et al.^139^	FFA	2.01	144.22	66.70	39.82	69.30	4.91	0.35	30 °C, –90 °C	-
Zhang et al.^148^	RM^c^	1.52	5.22	19.18	1.86	84.52	1.32	0.21–0.28	23 °C, 50 °C, 80 °C	-
	HWFFA^d^	2.11	N/A	N/A	44.55	N/A	9.16			
	B1 FFA^e^	1.89	153.49	22.02	31.98	63.20	6.74			
	B2 FFA^f^	1.86	175.00	19.96	33.44	57.07	6.10			
Liu et al.^149^	FFA 1	2.36	20.88	N/A	5.42	20.38	2.27	0.36	170 °F	-
	FFA 2	1.97	68.36	N/A	5.42	20.38	2.27			
Xiao et al.^26^	WG^g^	72.58	1.47	12.54	0.40	10.49	0.61	0.4	Ambient temperature	-
	CFA	44.40	18.70	0.8	1.40	18.50	3			
	Metakaolin	1.91	442.64	476.69	N/A	3098.5	21.07823			
Zhang et al.^31^	FFA	2.23	70.17	54.07	14.03	51.25	11.10	0.27	60 °C	Water to total solid ratio is used in this study
	Mine tailing	9.15	72.01	19.88	8.62	15.96	14.97			
Nasvi et al.^44^	FFA	1.58	241.50	120.75	17.25	40.25	3.99	N/A	23 °C, 30 °C, 45 °C, 60 °C, and 70 °C	L/S ratio = 0.4
Tian et al.^94^	Copper tailings	3.40	74.89	25.59	0.77	7.09	1.12	N/A	25 °C, 50 °C, 80 °C, 100 °C, and 120 °C	Copper tailings:coal fly ash:water glass:NaOH = 9:1:1.82:0.46 L/S ratio = 0.15
	FA	1.18	224.55	73.62	9.85	118.18	11.49			
Heah et al.^89^	Kaolin	1.49–1.52	1040–5000	26–33.33	>100	74.29–166.67	52–83.33	N/A	Ambient, 40 °C, 60 °C, 80 °C, and 100 °C	L/S ratio = 1
Chen et al.^150^	Metakaolin	1.23	206.73	298.61	335.94	-	119.44	N/A	20 °C, 40 °C, 60 °C, 80 °C, and 100 °C	L/S ratio = 1.33–1.47

a Class-F fly ash.

b Ground granulated blast slag.

c Red mud.

d Headwater Resources Inc. fly ash.

e Boral Material Technologies Inc. fly ash.

f Waste glass.

g Class-C fly ash.

### 3.1 System Chemistry

**Figure 3:**
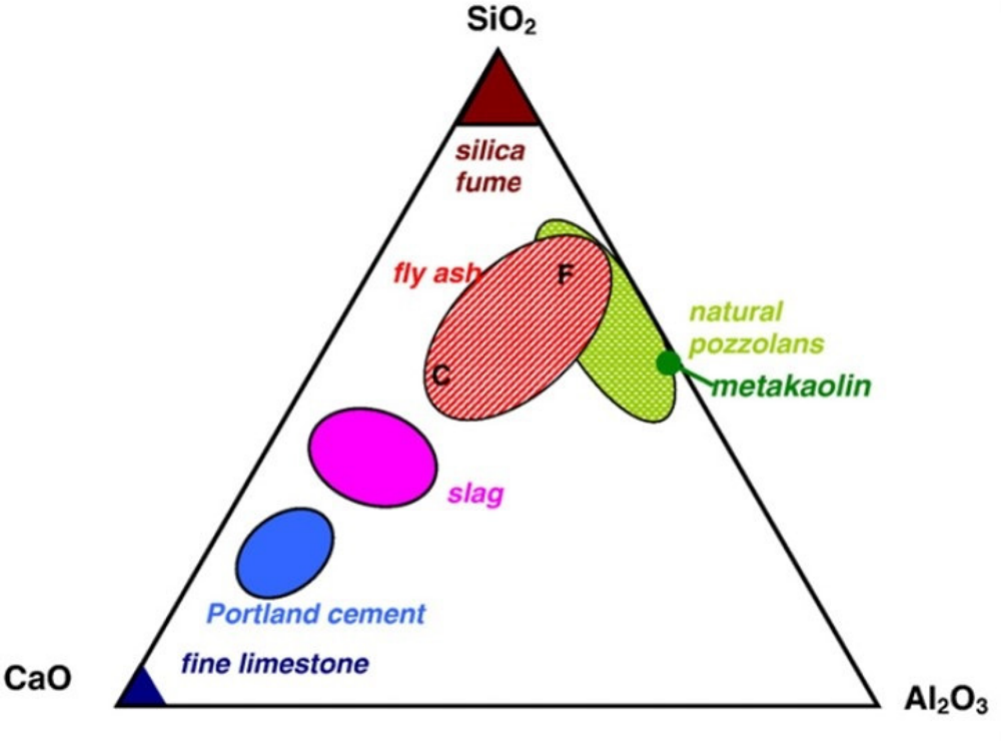
Ternary diagram of cementitious materials. Reproduced with permission from ref (51). Copyright 2020, Elsevier.

The chemical composition of the precursors is among the key factors controlling GP properties, especially within the acidic environment encountered during CCS operations.^43,146,151^ GP precursors are typically rich in silica and alumina, while amorphous materials may be preferred due to higher dissolution rates (and thus reactivity).^31,51,78^ The wide variety of materials that have been used in GP production complicates the study of the effects of chemical composition on the GP properties (see Figure 3 and Table 1). For instance, Xu and Van Deventer^132^ showed the potential of 16 natural aluminosilicates as precursors for GP synthesis, with different precursors leading to significant differences in the properties of produced GPs. In addition, the composition of each GP system encompasses both fully polymerized and unreacted phases, which results in further complications when studying the inherent binder phase.^75^ A further complication of the system is added through the widely different compositions of alkali solutions and additives that can be employed in the GP synthesis processes. To eliminate some of these complexities, elemental molar ratios within the system will be considered here as this can increase the accuracy of GP analysis and provide proper criteria for obtaining an optimum formulation for each GP system. Accordingly, this section briefly discusses the effects of Si/Al, Si/Na (or Si/K), Si/Ca, Si/Mg, and Si/Fe ratios on the behavior of GP systems and the optimum values for these ratios as reported in literature.

#### 3.1.1 Si to Al Ratio

The Si/Al ratio is a key factor controlling GP properties through its impact on the network connectivity (Si–O–T, where T is Al or Si) and the degree of Si(OH)_4_ and Al(OH)_4_ emancipation.^27,78^ In general, the Si content determines the condensation between Si–O–Si and Al–O–Si species and strongly influences late-age compressive strength of the material, while the Al content regulates a GP’s type of network formation, framework, and setting time.^29,152^ At low Si/Al ratios (i.e., higher Al contents), condensation of Al–O–Si species governs the system and results in the formation of polysialate structures, a matrix with larger grains, and thus lower compressive strength. In contrast, a high reactive Si content (i.e., lower Al content) leads to the formation of oligomeric silicates and rigid 3D networks of poly(sialate-siloxo)/poly(sialate-disiloxo) structures, with high compressive strengths at later ages but prolonged setting times, mainly due to the lower condensation rate between silicate species.^51,152^ Note that for systems with relatively high Ca content, Si/Al-dependent setting time trends are more ambiguous, as the Ca content also needs to be considered. While increased Al content leads to shorter setting times for conventional GPs with low calcium content (e.g., Class F fly ash), for systems with higher Ca contents (e.g., systems based on Class C fly ash), increasing both Si and Al content results in shortened setting times.^152,153^

**Figure 4:**
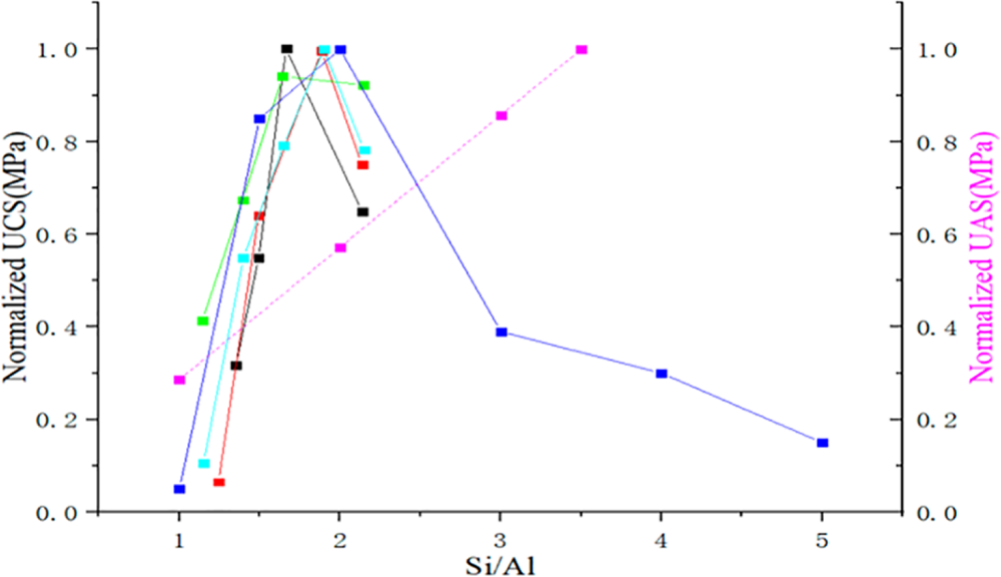
Normalized UCS and UAS (pink line) data reported the corresponding Si/Al ratios. Reproduced with permission from ref (42). Copyright 2021, American Chemical Society.

Accordingly, changing the Si/Al ratio of a GP system results in significant alterations in its workability, pore structure, density, and mechanical strength.^29,31,134,154,155^ The Si/Al ratio also influences adhesion properties of GP. Through analyzing the normalized unconfined compressive strength (UCS) and ultimate adhesion strength (UAS) reported in the literature, Rong et al.^42^ concluded that GP compressive strength increased with increasing Si/Al ratio up to 1.9 and then decreased if the ratio increased further, while the adhesion strength increased continuously with increasing Si/Al ratio (see Figure 4).

However, there are some disagreements about the impact of the Si/Al ratio on the compressive strength of fly-ash-based systems. While some research articles^80,148,156^ report an increase in mechanical strength of fly-ash-based GPs with increasing Si/Al ratio, others report insignificant,^157^ or even negative,^139,147,153^ effects of the Si/Al ratio. Similar contrary data have been reported regarding the impacts of the Si/Al ratio on the mechanical properties of metakaolin-based GPs,^158,159^ rock-based GPs,^27,50^ and others.^160^ The inconsistent results reported in the literature are sometimes ascribed to various processes employed in GP preparation.^42^ The dependency of a GP’s properties on its water content can also be an explanation for these dissimilarities. For instance, Yaseri et al.^161^ showed increased compressive strength with increasing Si/Al ratios for a metakaolin-based GP when precursor to alkali activator ratios were 1.2 and 1.4, while contrary results were obtained at higher ratios. Furthermore, at higher Si/Al ratios, unreacted particles may negatively impact mechanical properties by acting as defect sites, and the hindered evaporation of water at excessive silica contents may unfavorably impact the polymerization process.^40,51,119^ For fly-ash-based systems, differences in strength development may also stem from the chemical and physical heterogeneity of fly ash particles, as these heterogeneities may impact GP formation and properties.

**Figure 5:**
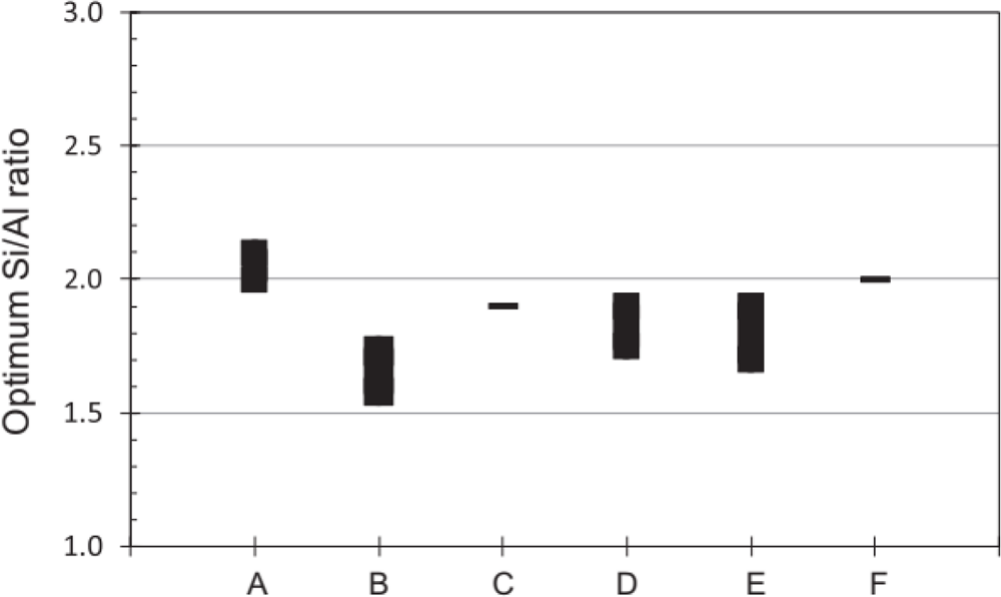
Optimum Si/Al ratios from different researchers for various sources (A = Fly ash, Kaolinite, Albite, B = Metakaolin, GGBFS, C = Metakaolin, D = Metakaolin, E = Metakaolin, F = Municipal solid waste incinerator fly ash). Reproduced with permission from ref (31). Copyright 2011, Elsevier.

Additionally, apparent inconsistencies in the literature results regarding the effect of the Si/Al ratio may have resulted from the ranges of Si/Al ratios employed and neglect of the existence of optimum Si/Al ratios. According to Zhang et al.,^31^ the optimum Si/Al ratio for different GP systems falls in the range of 1 to 3, but the exact ratio should be specified depending on the type and reactivity of the precursor(s) used. Higher optimum Si/Al ratios might be expected for precursors with higher reactivity, where alumina and silica species dissolve and are made available for geopolymerization more rapidly. Thus, when two GPs are produced with identical hardeners and precursors that are identical except in particle size, a higher optimum Si/Al ratio can be expected for the mix with the finer (and thus more reactive) precursor. Figure 5 presents a summary of the optimum Si/Al ratios reviewed by Zhang et al.^31^

One final consideration is that while the dissolution of silica is predominantly controlled by alkaline molarity alumina dissolution is governed mainly by (curing) temperature (see Section 3.3).^89,162^ This yields increased optimum Si/Al ratios at higher alkali concentrations and at lower curing temperatures.^77^

The research reviewed here, regarding the optimum Si/Al ratio of GP systems, thus shows that, while the Si/Al ratio may be a key component controlling in particular setting time and late-age mechanical properties, optimum Si/Al ratios are partly dependent on the properties of the precursor used (e.g., reactivity, particle size), system alkalinity, and curing temperature. In addition, the observed trends may be further complicated by the Ca content of the system. This thus shows a need for highly controlled experimental studies, especially when considering the high-temperature, low-pH environments that may be encountered by GPs used as wellbore sealants in CCS operations.

#### 3.1.2 Si to M and Al to M Ratios

The Si/M ratio (where M stands for Na + K) affects the properties of GPs mostly through controlling the rates of dissolution and gel formation.^57^ Alkali cations contribute to the liberation of Si(OH)_4_ and Al(OH)_4_ tetrahedra from precursors while also being necessary to balance the charges of the AlO_4_ tetrahedra. Overall, alkali metals (Na or K) govern the reaction extent and densification of GP microstructure, and their presence improves the compressive strength of the system.^26,80,126^ For instance, De Vargas et al.^163^ and Gao et al.^164^ demonstrated that lower Si/Na ratios reduce the setting time of GPs while improving their structure densification and compressive strength. Zhang et al.^148^ obtained similar increasing trends and concluded that for a constant Si/Al ratio of 2 nominal Si/Na ratios of 2.5–3.33 are proper starting points for the synthesis of GPs of different sources.^31^ For GP mortars produced from fused granite wastes, Tchadjié et al.^30^ obtained maximum compressive strengths at a Si/Na ratio of 0.65, while for clay-based GPs, MacKenzie^165^ obtained an optimum SiO_2_/Na_2_O ratio of 3.33.

The Al/M ratio is another controlling factor commonly stated in the literature. The optimal Al content in GPs is influenced by the requirement for cations to maintain charge balance of the system.^80,126^ Al/Na ratios commonly reported in studies fall in a range between 0.38 and 2.06.^31^ Low Al/M ratios can have negative impacts on GP properties that must be taken into consideration (see Section 3.4.1).^27,57,166^ For GP systems, the optimum Al/Na ratios reported in the literature are usually around 1.^31,51,59,79^ It is worthy to note that Al/M and Si/Al ratios determined by energy-dispersive X-ray spectroscopy (EDS) analysis are the values that control the compressive strength of GPs, rather than the starting molar ratios determined by X-ray fluorescence (XRF) analysis.^103,148^

#### 3.1.3 Si to Ca Ratio

While in recent studies much has been discussed regarding the role of Ca content in geopolymerization processes, key questions remain that should be addressed through further laboratory research. A positive impact of Ca on the strength development of GPs has been reported and ascribed to the formation of C(−A)–S–H gels filling voids in the GP system, leading to enhanced mechanical durability.^81,85,153,167^ Furthermore, in cementitious materials with CaO contents above 20%, accelerated hardening and improved mechanical durability have been reported, as a result of extra nucleation sites provided by increased Ca content.^31,65,84,88,133,168,169^ While C(−A)–S–H gels can form at both low and high alkali contents as long as sufficient Ca is present, at high pH values, Ca dissolution (and thus C(−A)–S–H formation) is inhibited by the OH^–^ ions in solution, until sufficient OH^–^ ions have been consumed in the polymerization reactions.^31^ Rapid dissolution of calcium then results in the formation of Ca(OH)_2_, yielding increased numbers of nucleation sites. The accelerated formation of C(−A)–S–H gels also causes a further drop in pH and thus a reduction in dissolution rates. This dual behavior complicates our understanding of the kinetics of calcium-containing GP formation, even for low Ca-content precursors such as Class F fly ashes.^88^

**Figure 6:**
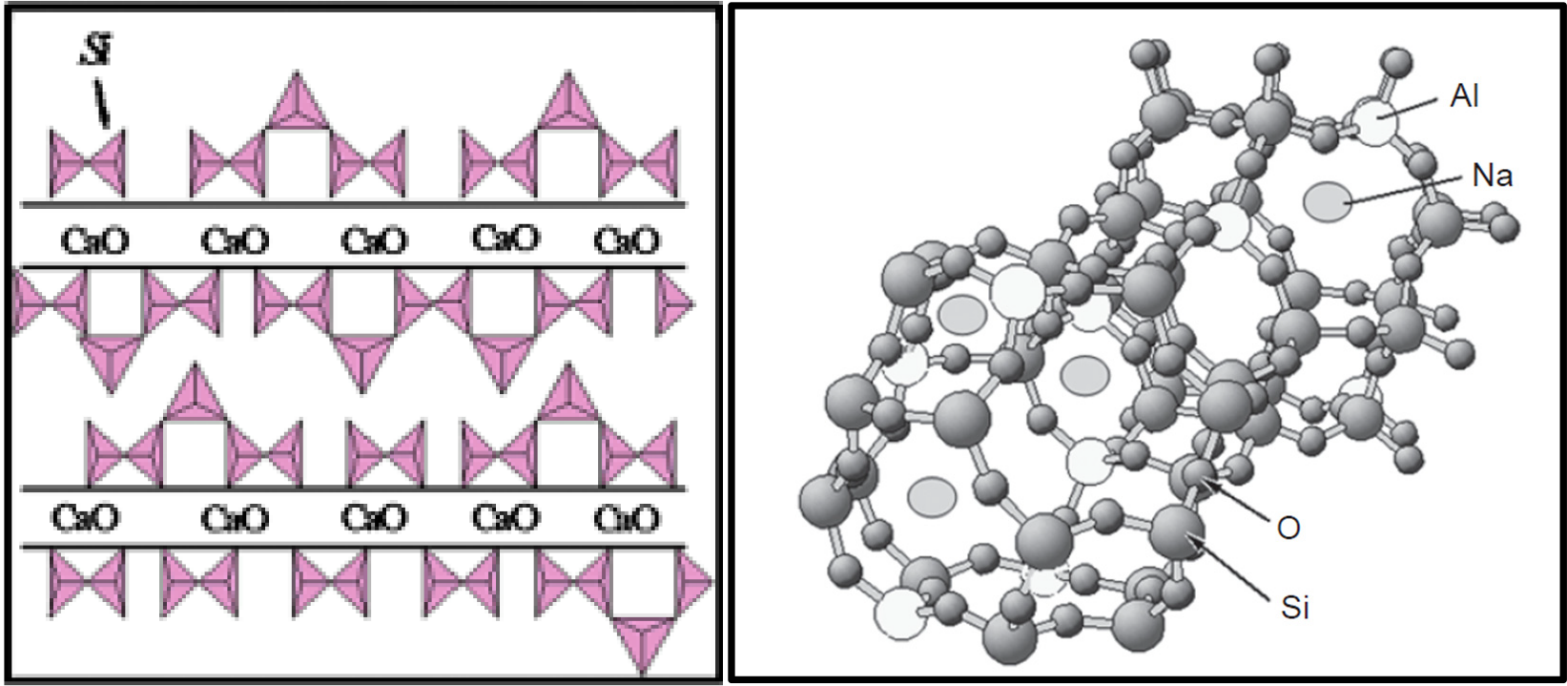
Schematic representation of the linear structure of C(−A)–S–H gel (left) (Reproduced with permission from ref (168). Copyright 2010, John Wiley and Sons) and 3D structure of N–A–S–H gel (right) (Reproduced with permission from ref (79). Copyright 2014, Elsevier).

Furthermore, it is widely acknowledged that the active Ca content of aluminosilicate materials has a strong control over the nanostructure and durability of alkali-activated setting materials exposed to low pH and/or CO_2_-rich conditions, such as those expected in CCS operations. Though the alkali activation of low-calcium aluminosilicates mainly leads to the formation of a highly cross-linked N–A–S–H structure of gel polymers with structures similar to those of zeolites, the alkali activation of materials with higher Ca contents leads to the formation of tobermorite-like C(−A)–S–H gels.^62,145,170^ While compositionally similar, there are fundamental differences in structure between these two gels that lead to hugely different material properties. C(−A)–S–H gels consist of linear chains of linked silicate tetrahedra where a central CaO layer is surrounded by repeating three-unit components, while N–A–S–H gels consist of an amorphous to semicrystalline 3D structure of silica-tetrahedra with a random distribution of aluminum replacing silicon, which is created through nucleation and growth stages (see Figure 6).^23,50,140,168^ While the denser microstructure of C(−A)–S–H gels compared to N–A–S–H gels leads to higher mechanical strengths,^103,171^ C(−A)–S–H gel structures are more vulnerable than N–A–S–H gels to chemical attack when exposed to fluids with low pH and/or high CO_2_ concentrations, as the Ca present in the structure of these gels is vulnerable to consumption in carbonation reactions (Section 3.4).^35,172−175^

While GPs can be synthesized with no Ca in the system, such GPs require curing at elevated temperatures of at least 50 °C. This restricts their practical application in the field of civil engineering but not necessarily in wellbore construction.^65,168^ In GP systems based on precursors that do not contain Ca, Ca is commonly incorporated as an additive to eliminate this need for elevated curing temperatures, for example, through the addition of OPC, GGBFS, class C fly ash, calcium hydroxide, calcium carbonates, calcium aluminate cement, or natural calcium silicate materials.^65,84,117,176−179^ In fact, many argue that the coexistence of N–A–S–H and C(−A)–S–H gels leads to the reduced porosity/permeability, reduced water adsorption, increased density, stronger networks benefiting from composite binding phases, and higher compressive strengths of the GP system.^26,31,35,84,180^ For instance, the positive impacts that Ca can have on GP gel structures is demonstrated by Yang et al.,^181^ who reported the presence of amorphous homogeneous N–C(−A)–S–H in fly-ash-based GPs modified with slags, where calcium contributes to the formation of a more compact microstructure and consequently higher compressive strengths compared to unmodified fly-ash-based GPs.^121,122^

#### 3.1.4 Si to Mg and Si to Fe Ratios

Sufficiently high MgO contents in a GP system may contribute to reduced setting times and increased mechanical strength, as MgO leads to the formation of OH^–^, increasing alkalinity and thus enhancing reaction kinetics.^116,134,182,183^ Furthermore, higher MgO-content can result in the precipitation of expansive hydrotalcite (Mg_6_Al_2_(OH)_16_CO_3_·4H_2_O)-like phases, reducing the pores size, leading to structural densification, and thus yielding improved mechanical durability of the systems.^69,116,184−187^ The improved density and mechanical performance of GPs containing MgO are also sometimes ascribed to a filler effect of MgO.^188^ However, other studies have reported detrimental impacts of magnesium impurities (such as MgSO_4_, burnt magnesia, MgCl_2_, etc.) on the mechanical performance of GP/AAM systems.^79,182,186,189,190^ Such negative impacts may be due to the formation of Mg(OH)_2_, which results in increased gel volume, local volumetric expansion (up to 118%), and local stresses that can generate microcracks, thus leading to reduced mechanical strength. In addition, interactions between Mg^2+^ and N–A–S–H gel structures can lead to the production of Mg–A–S–H (or N–Mg–A–S–H) gels, which may have lower compressive strengths than N–A–S–H gels.^134,186,189,191,192^ Likewise, Ismail et al.^190^ argued that the reduced mechanical strength of slag-based systems in the presence of Mg^2+^ ions was caused by the breakage and weakening of Ca bonds and destroyed C(−A)–S–H gel structures.

MgO is also well-known as an efficient shrinking and reducing admixture for the OPC applications. However, studies regarding the impacts of MgO on the shrinkage of GPs are still in testing or development phases, with literature findings mainly showing that highly reactive MgO, with a completed hydration process in 1 day, leads to the reduction of drying shrinkage but also to the development of severe cracks at dry conditions. In contrast, using moderately reactive MgO, which completes it hydration after one month, results in improved soundness but only a moderate shrinkage reduction.^51,182,186,187,193^ It is worth noting that there is also limited evidence for MgO improving the carbonation resistance of a GP, as it leads to a reduced penetration depth of CO_2_. This is ascribed to the precipitation of an expansive amorphous hydrotalcite-like phase and associated reduction in porosity and thus permeability of the system as well as to magnesium carbonate precipitation which impedes further CO_2_ penetration and inhibits carbonation of the C(−A)–S–H gels.^69,184,194−196^ However, the use of excess amounts of MgO may result in increased porosity due to the relatively low bulk density of the hydrotalcite-like phases (2.0 g/cm^3^) compared to C(−A)–S–H (2.23 g/cm^3^) and can thus lead to reduced resistance to volume alterations.^116,187^

Only limited research exists regarding the effect of Si/Fe ratios on the properties of GPs.^78,166,197^ In general, high concentrations of ferric oxide, apart from its impacts on the color of GPs, can give higher specific gravity, enhanced thermal conductivity, and increased thermal expansion of the setting material while also impacting the morphology of GPs after curing at elevated temperatures. For these reasons, Fe is considered incompatible with GPs at elevated temperatures (above 800 °C).^49,51,198^ GPs with higher iron oxide contents also show lower acid resistance, even when compared to OPCs.^199^ With regards to mechanical properties, some recent studies have reported higher compressive, flexural, and tensile strengths of iron-bearing GPs, which has been ascribed to the filler effects of iron oxides and the combined action of polysialates, iron-silicates, and ferro-sialates (Fe(−Al)–S–H).^166,188,200−204^ Other studies, however, have shown that iron can negatively impact GP properties through the rapid precipitation of Fe species due to the higher atomic size of Fe compared to Si or Al, causing more rapid consumption of OH^–^, which in turn results in deceleration of the dissolution of the remaining precursor and inhibition of the geopolymerization and thus reduced strength of the system.^51,185,205^ However, as shown by Davidovits,^98^ these negative impacts of elevated Fe contents on GP properties may be suppressed, as they achieved improved mechanical durability of GPs even with extremely high quantities of iron. Therefore, the role of Fe has not been fully understood, especially with respect to the CO_2_ resistivity of the Fe-containing systems, and further research is required.

Table 2 presents the results of studies attempting to explore the optimum molar ratios for GP systems prepared from diverse types of precursors. Optimum values are selected based on the resulting GP’s compressive strength, as the most used indicator for assessing the success of GP’s technology.^133^ As shown, the proposed values significantly vary depending on the composition and type of raw materials. Consequently, independent studies should be carried out for each new GP formulation to determine the specific optimum value of the given formulation.

**Table 2:** Brief Summary of the Optimum Molar Ratios of GP Systems Proposed in the Literature

Main precursor(s)	Author(s)	Alkali metal	Alkali concentration (wt %, M)	SiO_2_/Al_2_O_3_	SiO_2_/M_2_O	H_2_O/M_2_O	W/S ratio	L/S ratio	Curing temperature	Compressive strength (MPa)	Comments
Metakaolin	Duxson et al.^40^ and Duxson et al.^136^	Na, K	-	3.8	3.8	11	-	-	20 h at 40 °C	75	The maximum strength is the result of 7 day UCS.
	Steveson and Sagoe-Crentsil^135^	Na	-	3.5–3.8	2.92–3.17	12	-	1.44–1.51	2 h at 85 °C, followed by one-week curing in refrigerator	48	The maximum strength is the result of 7 day UCS.
	De Silva et al.^152^	Na	-	3.4–3.8	3.4–3.8	13.6	-	-	72 h at 40 °C	23	The maximum strength is the result of 24 h UCS.
	Cheng and Chiu^119^	K	-	3.16–3.46	1.32	33.33	-	-	3 h at 60 °C, followed by 28-day curing at ambient temperature	70	The maximum strength is the result of 28 day UCS.
	Yaseri et al.^161^	Na	14	4.8	7.143	10.31	0.25	-	24 h at 60 °C	68	The maximum strength is the result of 7 day UCS.
	Rowles and O’connor^206^	Na	-	5	3.84	-	-	-	24 h at 75 °C, followed by 7 day curing at ambient temperature	64	The maximum strength is the result of 7 day UCS.
	Zhang et al.^207^	K	-	4.5	5.625	5	-	-	28 days at 20 °C and 95% relative humidity (RH)	34.8	The maximum strength is the result of 28 day UCS.
	Yunsheng et al.^208^	Na	-	5.5	5.5	7	-	-	28 days at 20 °C and 95% RH	34.9	The maximum strength is the result of 28 day UCS.
Fly ash	Temuujin et al.^209^	Na	-	7	7	-	0.35		Ambient	3.9	Water content is presented in wt %. The maximum strength is the result of 7 day UCS.
	Pavithra et al.^140^	Na	16	6	-	-	-	-	24 h at 70 °C, followed by 14 day curing at ambient temperature	48	The maximum compressive strength is obtained at the Na_2_SiO_3_/NaOH ratio of 1.5. The maximum strength is the result of 14 day UCS.
	Hardjito et al.^139^	Na	14	2–3.5	-	-	0.174	0.35	24 h at 60 °C, followed by 7 day curing at ambient temperature	67.6	The maximum compressive strength was obtained at Na_2_SiO_3_/NaOH ratio of 2.5 and is the result of 7 day UCS.
											The proposed optimum SiO_2_/Al_2_O_3_ ratio is based on precursor chemistry.
	Wang et al.^48^	Na	-	1.5	-	-	-	0.254	12 h at 80 °C, followed by 3, 7, 28, and 90 days curing at ambient temperature	36.8 (3 days)	UCS tests were conducted after 3 day, 7 day, 28 day, and 90 day curing times.
										39.2 (7 days)	
										45.5 (28 days)	
										46.3 (90 days)	
	Timakul et al.^210^	Na	-	2.65	-	7	-	-	96 h at 75 °C, followed by 28 days curing at ambient temperature	40	Na_2_SiO_3_/NaOH ratio is 1. The maximum strength is the result of 28 day UCS.
	Chindaprasirt et al.^153^	Na	-	3.2–3.7	4.76	11.45	-	-	24 h at 60 °C	64	CaO/SiO_2_ = 0.69
											The maximum strength is the result of 24 h UCS.
	van Jaarsveld et al.^211^	K	-	1.75	0.877	-	-	0.31	12 h at 70 °C, followed by 14 days curing at ambient temperature	34	UCS tests were conducted after 14 day curing time. Longer curing times at 70 °C resulted in weakened structure of GPs due to additional water loss and cracking.
	Zhang et al.^148^	Na	-	4	2.5	-	0.27	-	Up to 28 day curing at 23, 50, and 80 °C and different RH levels	13.5	EDX Si/Al = 3.6
											EDX Na/Al = 1.6
											The maximum strength is the result of 28 day UCS.
	Steveson and Sagoe-Crentsil^212^	Na	-	3.9	3.9	10	-	0.92	2 h at 85 °C, followed by 1 week in refrigerator	47	The formulations had higher L/S ratios than those used by previous researchers. The maximum strength is the result of 7 day UCS.
	Somna et al.^131^	Na	14	2.81	2.81	5.94	-	-	Ambient	25.5	The maximum strength is the result of 60 day UCS.
Rock-based	Nadeem et al.^27^	Na	-	22.84	4.05	-	-	0.22–0.3	24 h at 70 °C, followed by 2 h curing at 220 °C	22	Si/Ca = 2.911
											SiO_2_/MgO = 2.59
											SiO_2_/Fe_2_O_3_ = 6.63
											Na_2_SiO_3_/NaOH ratio is 2.57
	Tchadjié et al.^30^	Na	-	5.875	0.47	-	-	-	Ambient	40.5	Alkali fusion with NaOH was used to increase the reactivity of granite waste. UCS tests were conducted after 28 day curing time.
Others	Xu and Van Deventer^169^	K	-	4.2	0.172	8.55	-	0.294	24 h at 40 °C, followed by 6–27 days curing at ambient temperature	45	Kaolinite, albite, and fly ash were the precursors. UCS tests were conducted after 28 day curing time.
	Kaya et al.^188^	Na	-	3–5	0.77–2	-	-	0.45	24 h at 110 °C, followed by 28 days curing at ambient temperature	14.5	Zeolite and kaolinite were the precursors. The maximum strength is the result of 28 day UCS.
	Xiao et al.^26^	Na	5	6.076	-	-	-	0.4	Ambient	34.5	Waste glass, fly ash, and limestone powder were the precursors. The maximum strength is the result of 60 day UCS. Si/Ca = 2.91
	Kani and Allahverdi^213^	Na	-	0.69	0.75	8.15	-	-	Ambient	50	Pumice-type pozzolans were used as precursors. The maximum strength is the result of 28 day UCS.

### 3.2 Water Content

Water content strongly impacts the key properties of GPs in both slurry and hardened states, such as density, viscosity, setting time, microstructure, porosity, mechanical strength, and bonding strength,^63,145,158,203,214−216^ While a minimum water content is thus required to act as electrolyte, and for emplacement of any GPs increasing water contents mostly have negative effects on the key properties of cured GPs.^217^ Increased water content adversely affects the dissolution and polycondensation stages through reducing the alkalinity of the system. This effect can be amplified during ongoing geopolymerization, as geopolymerization releases additional water.^87,217^ In addition, increased water content has a detrimental impact on the adhesion properties and dimension stability of GPs. These effects have been shown to be of greater significance at lower Na/Al ratios.^42^

Accordingly, laboratory studies have shown that at constant Si/Al ratio higher water content results in increased porosity and reduced mechanical strengths.^87,139,203^ One additional mechanism by which high water content can reduce a GP’s mechanical strength is through the increased mobility of Na cations, which results in the destabilization of AlO_4_, breakage of Al–O bonds, and the formation of AlO_3_ units near nanovoids, that can then act as preferential sites for fracturing.^127^ Thus, when developing a GP, a balance must be found between the minimum water to binder (w/b) ratios required for workability, density, and slurry viscosity and the maximum w/b ratios that will still result in the desired properties of the hardened GP.^218^ To support this, the water demand can be reduced using commercial water-reducing agents (i.e., dispersants).^139,219,220^ Moreover, there is some experimental evidence revealing the prolonged setting time and improved compressibility of GP systems through the addition of chelators to precursors, where the chelator type determines the degree of prolongation.^74,155^

Finally, it should be noted that when discussing a GP’s water content it is worth mentioning that, in contrast to OPC-based binders, GPs do not consume water but produce water because of polycondensation reactions.^19,67^ Additionally, it is important to differentiate among the various states of water in the GP structure. Water can be present in a GP structure as (a) evaporable water, including free water present in larger pores, and physically bound water tightly held into ultramicropores of hydroxylated silica and (b) nonevaporable water (i.e., chemically bound water).^214,221^ The chemically bound water is strongly linked to the strength of GP species, and its presence shows a high extent of cross-linking in the gel phase.^222^ A recent study by Park and Pour-Ghaz,^214^ investigating the role of water content in metakaolin-based GPs, showed that the state of water within a GP structure was influenced by the ratio of NaOH to metakaolin (NaOH/MK), where higher NaOH/MK ratios led to the production of additional water, while at lower NaOH/MK ratios, water was physically or chemically bound within the GP structure. In addition, in an earlier study, Duxson et al.^221^ showed the presence of greater quantities of physically bound water at higher Si/Al ratios, where the probability of silica condensation is higher, while GPs with lower Si/Al ratios contained a zeolitic phase in which water is tightly absorbed within the cage-like gel structures.

### 3.3 Curing Conditions

The influence of curing conditions on the properties and characteristics of GPs has been extensively studied in laboratory experiments. These conditions include curing temperature, curing time, and relative humidity (RH).^46,75,144,223−225^ Failure to implement proper curing treatments can lead to negative outcomes such as reduced mechanical strength and increased vulnerability to CO_2_ attack, chloride ingress, and corrosion of steel embedded in the GPs (see Section 3.4).^226,227^

As the reactions that lead to geopolymerization are enhanced as temperature goes up, curing at elevated temperature is commonly required to obtain desired properties.^44^ At low curing temperatures, aluminosilicate dissolution rates are limited, thus restricting the geopolymerization process due to the limited availability of Si and Al. Additionally, excessive water content may adversely affect the strength development of the material under low-temperature conditions (see Section 3.2).^53,89,103,162^ At higher curing temperature, as dissolution kinetics are enhanced, hardening is also accelerated so that higher effective strengths can be achieved more quickly. However, at the same time, higher temperatures also shorten open times, during which the slurry can be pumped, emplaced, and consolidated.^3,28,44,53,86,162,228,229^ Furthermore, prolonged curing at high temperatures can lead to partial water evaporation, increased pore size, and microcrack development, thus resulting in higher permeability of the GP and increased susceptibility of the system to chloride ingress and CO_2_ attack.^44,46,89,94,139,148,228,230−232^ The increased evaporation of water from the capillaries can also inhibit the creation of a dense and durable GP by impeding the formation of N–A–S–H gels. This is because a significant portion of aluminosilicate dissolution occurs in water-filled capillaries.^227,233^ Moreover, excess gel formation at high curing temperatures can hinder the further dissolution of Si and Al species.^51^ As noted previously (Section 3.1.1), Si-dissolution kinetics are less sensitive to temperature than Al-dissolution kinetics, meaning that at elevated temperatures more Al is available (lower Si/Al ratio), which, in turn, can potentially impede compressive strength development.^89,162,227,233^ The rapid increase in solution viscosity during the onset of polycondensation and subsequent reduction in ion mobility, along with dehydration-induced shrinkage caused by gel contraction before transitioning into a more semicrystalline structure, are additional outcomes of prolonged curing at elevated temperatures that can negatively impact the ultimate strength and other properties of GPs.^44,53,86,89,103,148,162^

The negative impacts of elevated curing temperatures on GP properties can be mitigated by increasing the alkalinity of the system and adding waterglasses (soluble silicates). These measures enhance precursor dissolution and increase the Si/Al ratio, leading to accelerated kinetics that facilitate the transformation of N–A–S–H gel into zeolitic structures and the formation of semicrystalline and polycrystalline phases. This ultimately results in higher compressive strength.^53,103,227^ In addition, precuring preceding the conventional curing treatment has been widely recognized as an effective method to enhance the strength development of GP materials, particularly in laboratory-scale research. Among various precuring techniques, preheating by microwaves, with its uniform and rapid heat curing nature, has also been found to enhance dissolution rates of Si and Al species, increased material density and homogeneity, and ultimately higher strength of GPs. While autoclaving and steam precuring methods have also been reported to contribute to the strength development of GP systems, the nonuniform characteristics and slower heating rates associated with steam precuring can lead to larger pore size distribution and lower material densities compared to other precuring techniques.^51,86,226^

Accordingly, numerous studies have shown the existence of optimum curing temperatures, at which a specific composition will display optimal mechanical, chemical, and physical properties.^94,223,231^ Curing the samples at optimum values leads to increased percentage of Si sites in N–A–S–H and C(−A)–S–H gels, more incorporation of [AlO_4_]^5–^ tetrahedra into the backbone of [SiO_4_]^4–^ tetrahedra, and increased compressive strength of the GP system.^94^ Example optimum curing temperatures reported in the literature are 55–90 °C for fly-ash-based GPs,^9,86,148,230,234^ 40–80 °C for waste-glass-based GPs,^26,112^ 80 °C for metakaolin-based GPs,^235^ and 80–90 °C for copper-tailing-based GPs.^53,94,234^ Differences in the optimum temperature of GP materials, even when using the same precursor, are largely caused by the intricate relationship between curing temperature, curing time, and the reactivity and composition of the precursor material.^44,79,86,148^ When all other factors remain constant, highly reactive precursors, such as metakaolin, tend to complete the geopolymerization process within shorter curing times and/or at lower curing temperatures compared to less reactive sources, such as red mud and rice husk ash.^51,86,148,226^ However, Farhan et al.^51^ have demonstrated that the differences in strength and stiffness tend to diminish over time.

Proper control of RH during the curing process is also crucial for achieving volume stability and pore size control and preventing water loss, shrinkage, and crack formation in GPs.^103,236^ When the RH is low, the dry and self-shrinkage of particles with large wetting surface areas, such as microsilica and metakaolin, can adversely impact the strength development of GPs. Conversely, RH levels above the optimum have been shown to reduce the strength of GP materials due to the increased leaching of dissolved species out of the system as well as the large expansion of the material.^51,215,227,236^ Furthermore, Oderji et al.^237^ suggested that strength reductions observed at excessive RH conditions can be attributed to the slower water release that occurs at all stages after dissolution, causing delayed hardening of the material. Although some limited deviations have been reported in the literature,^215^ there is a general consensus that curing at an optimal RH is critical for the effective development of GP materials.^51,103,215,226,227,236^ It should be noted here, however, that under downhole conditions, in water-filled or humid geological reservoirs, the concerns for water evaporation are not relevant.

Regarding the impacts of curing (exposed) water composition, recent studies revealed a decline in the rate of alkali leaching at high salinity conditions, expected in CO_2_ storage, which is favorable for GP systems and results in lower strength reductions compared to those of the OPCs, as discussed in Section 4.2. However, the long-term compressive strengths of GPs cured with saline water may be reduced due to salt crystallization inside the GP structure, which may induce internal stresses that lead to reduced strength.^2,7,8,67,68,238^ It has also been shown that high salinity curing increases the Young’s modulus of GPs.^2,232^

### 3.4 CO_2_ Exposure

Despite the rapid advancement of GP technology and a small number of research articles demonstrating the acceptable mechanical durability of the systems exposed to high CO_2_ concentrations (and humidity), important scientific and practical concerns remain that need to be addressed.^32,45,46,239^ The following sections discuss the effects of efflorescence and carbonation on GP systems, as these are known as the main mechanisms behind GP deterioration in the presence of CO_2_ and water and may impact the practical application of GP systems in CCS operations.^48,75,126^

#### 3.4.1 Efflorescence

**Figure 7:**
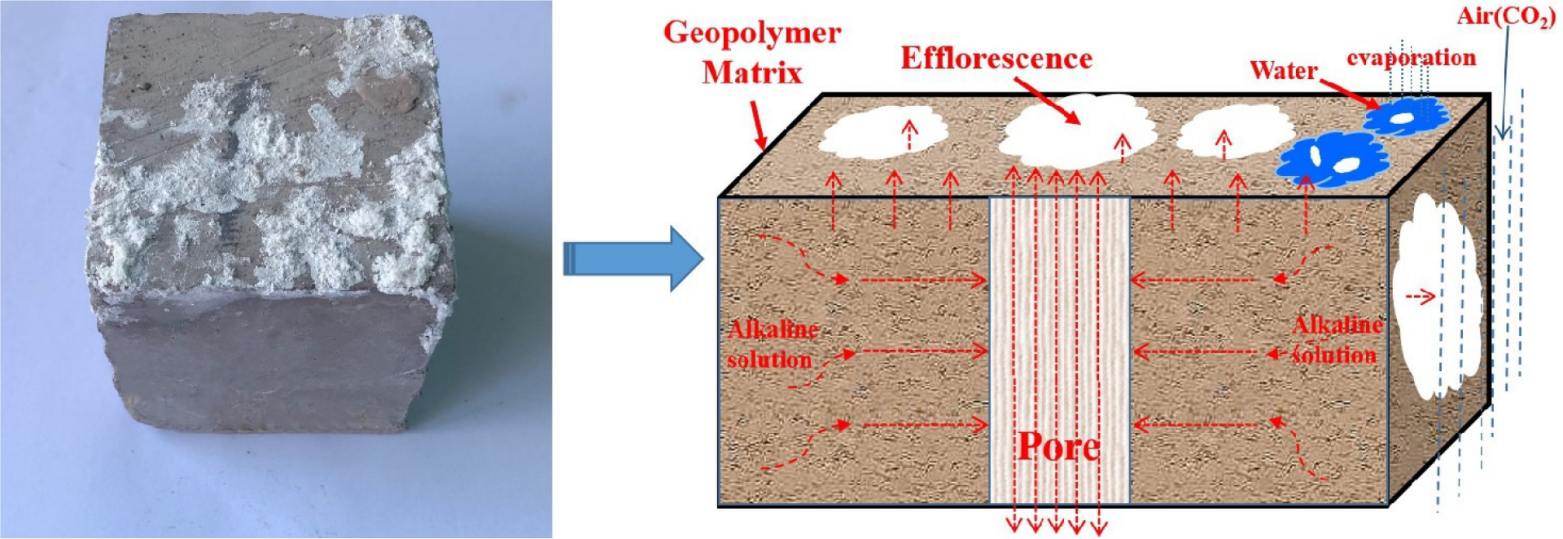
Schematic diagram of the GP efflorescence mechanism. Reproduced with permission from ref (48). Copyright 2020, Elsevier.

Efflorescence, the precipitation of white salt deposits on the external surface of GPs, occurs when excess unreacted alkalis, also known as free alkalis, are exposed to CO_2_ and humidity.^43,48,50,70,128^ GPs may contain such free alkalis due to their formulation, or alkalis may be taken up from the environment, for example from saline fluids.^37^ While efflorescence on the outer surface of concrete structures is merely unsightly, under conditions where such efflorescence takes place, precipitation of carbonated alkalis such as sodium carbonate heptahydrate (Na_2_CO_3_·7H_2_O) within capillary pores can also affect the internal structure of the GP (see Figure 7).^37,48,121^

While efflorescence is also a commonly observed phenomenon in OPC-based materials, their efflorescence is known as a superficial problem limited to discoloration that otherwise does not affect the integrity of the material.^121,138^ In GP systems, however, due to their higher content of alkali metals and the relatively free nature of such alkali ions (especially Na^+^) in the GP framework, efflorescence might be a formidable challenge that causes significant increases in permeability and potentially internal stresses as well as reductions in compressive strength, tensile strength, and durability of the system.^48,66,79,229,240,241^ Despite these significant impacts on GP integrity, efflorescence and the mechanisms behind it have received only limited attention in the literature, and so far, the evaluation of efflorescence effects on GP properties has relied heavily on findings obtained from experiments on OPC-based materials.^37^ In GP systems, key parameters controlling the degree of alkali leaching include permeability, system chemistry, water content, and aging conditions.^37,50^

- Permeability: Higher permeability results in increased rates of fluid transport through the GP and thus in increased potential for leaching and transport of cations out of the system. In this manner, higher permeability can thus result in reduced durability of the GP system.^48,50,51,126^ Using certain additives, such as nanosilica, calcium carbonate, or slags, can improve the densification of GPs, reducing permeability and thus impeding leaching and efflorescence.^48,50^ Alternatively, wettability alteration toward a more hydrophobic state has also been suggested to reduce the relative permeability of CO_2_, the leaching rate of alkali ions, and the degree of efflorescence.^48^ The pore structure of GPs is discussed in more detail in Section 4.
- Chemical composition: One of the major chemical factors affecting the rate of efflorescence is the Al/M ratio, as this directly impacts the availability of the alkali contained in the system. While in OPC-based materials, the foremost measure to avoid efflorescence-related challenges is reducing their alkali (M) content, and a greater deal of complexity is expected in GP systems.^37,48,79^ In an ideally stoichiometric chemical composition, one alkali cation supplements the charge of one four-coordinated Al^3+^, implying there may be an optimum Al/M ratio of around 1.^31,50,66^ However, as the geopolymerization process commonly does not reach a complete reaction of the components, lower alkali contents, i.e., higher Al/M ratios, that are stoichiometrically matched to the actual degree of geopolymerization may reduce efflorescence.^50,63,75,127,131,145,148,224^
- Thus, as an alternative to lowering the alkali content, increasing the Al content may also reduce the potential for efflorescence.^26,79^ For instance, in the work conducted by Wang et al.,^48^ fly-ash-based GPs with a Si/Al ratio of 1.5, benefiting from the highest content of [AlO_4_]^5–^ structures, showed the least degree of efflorescence between the samples with Si/Al ratios ranging between 0 and 2.5. However, according to Loewenstein’s principle of aluminum incompatibility, Al–O–Al bonds cannot be produced during the synthesis of GPs. Too low Si/Al ratios thus result in the production of unreacted [SiO_4_]^4–^ and [AlO_4_]^5–^ tetrahedra, a reduced degree of geopolymerization, and an increased potential for efflorescence.^37,41,48,242^ Furthermore, higher Al contents, over a certain optimum value, can also lead to increased imprisonment of cations and, subsequently, increased rate of efflorescence.^37^ Hence, accurate optimization of the Si/Al and Al/M ratios is key to developing GPs with the required mechanical properties and high chemical durability. When considering activation, the presence of waterglass promotes the early age potential of efflorescence, while it fairly reduces the long-term rate of efflorescence. In fact, waterglass has a limited influence on the overall potential of efflorescence.^121^ Zhang et al.^75^ showed that, at ambient curing temperature and with equal alkalinities, silicate-activated GPs exhibit greater rates of efflorescence compared to NaOH-activated compositions. Here, we should also note that Na^+^ cations, due to their smaller size and higher mobility, can cause increased efflorescence compared to larger K^+^ cations.^26,75,79^
- The addition of calcium aluminate cement as an admixture to the precursor mix can lead to the formation of N–A–S–H gels with robust cross-linked structures, yielding significant reductions in the mobility of alkalis and thus reduced efflorescence.^66,112^ Similar effects of slag admixtures on the rate of efflorescence have also been attributed to the high Ca content of these materials, but some unresolved questions remain regarding the role of Ca content in controlling the mobility of alkali cations.^26^ For instance, while Zhang et al.^75^ assert that slag admixtures can delay the onset of efflorescence even if they cannot prevent efflorescence entirely, Yao et al.^243^ reported a significant degree of efflorescence in fly-ash-based GPs modified with partial substitution of slags. Potential explanations for these different results could be the effects of temperature and the role of geochemistry of the used slags (i.e., interactions with Ca content of precursors).^26^
- Water content: While water molecules contribute to dissolution and repolymerizations of Al–O and Si–O monomers, a portion of water does not participate in these reactions and gradually evaporates throughout the synthesis process. As this aids the migration of unreacted alkalis through the GP microstructure, reducing water loss rates of GPs can help limit efflorescence.^48,126^ Despite such a significant role of water content in the occurrence of efflorescence, governing mechanisms are not well understood, and further research is required on this topic.^127^
- Curing conditions: It has been reported that curing samples at elevated temperatures (up to an optimum curing temperature) result in partial crystallization and reduced pore size volumes of GPs.^75,79,126^ Curing samples in water can also leach the excess Na^+^ from the surface of the material.^50^ In addition, hydrothermal curing accelerates alkali reactions and enhances local crystallization and reorganization of N–A–S–H gels.^75,126,244^ Therefore, curing under such conditions may reduce the intensity of efflorescence.
- Currently, the lack of agreement on standard techniques for evaluating the potential of efflorescence from a GP system is a major gap, complicating the interpretation of measurements on efflorescence reported by different researchers.^48^ Moreover, GP stability within the CO_2_-rich environment of CCS wellbores and its relationship with the degree of efflorescence have not been explored thoroughly.

#### 3.4.2 Carbonation

Carbonation is a physicochemical process which results from the interactions between surface-to-interlayer parts of the cementitious material, diffused CO_2_, and water.^50,71−73,77,176^ Diffusion of CO_2_ into the alkaline GP leads to a decrease of the pH of the pore fluid solution, triggering reactions that can profoundly affect the microstructure, chemical composition, porosity, permeability, mechanical strength, and durability of the hardened material.^5,120,176^ Under specific conditions, this can also lead to enhanced efflorescence.^77^ Nevertheless, while standardized tests have been developed to analyze the impacts of carbonation on OPC structures, carbonation mechanisms in GPs are to a large extent unknown and in need of further investigation.^72,245^

In the case of the OPC, the pH of the pore fluid is maintained at about 12.5 by the presence of portlandite (Ca(OH)_2_). When OPC is exposed to acidification, due to CO_2_ ingress or other mechanisms, this portlandite is dissolved, leading to a reduction in the pore fluid pH to values lower than 9. This in turn leads to an increased level of chemical degradation of structural phases, such as the C(−A)–S–H gel. In GP systems, however, carbonation mechanisms are different, as the main sources of alkalinity are the activator solutions used, based mainly on NaOH and KOH. A reaction of these components with CO_2_ produces Na_2_CO_3_ and K_2_CO_3_, which can induce a carbonate–bicarbonate phase equilibrium and reduce the alkalinity of the system (to pH values around 10–10.5).^5,14,34,72,79,174,245^ Due to the presence of free alkalis and the high reactivity of these unreacted species, GPs are also prone to carbonation; however, some studies have demonstrated a much lower impact of carbonation on GP properties compared to OPC.^32,44,45,71,72,176,177,246,247^ This might be due to the fact that, in OPC, both C(−A)–S–H and portlandite can be carbonated, while in GP systems, C(−A)–S–H gel (if present) is the phase that can be directly carbonated.^248^

The effects of carbonation on GP integrity can be approached from two different angles. While carbonation can be considered purely as a detrimental process, it can also be incorporated into the GP system as a process that improves GP properties. In the former case, GP formulations seek to improve the resistance of the system against carbonation, whereas in the latter, formulations may seek to enhance the potential for carbonation. GPs may even be exposed to elevated concentrations of CO_2_, as these can lead to higher mechanical strengths and lower porosities.^37,176^

When considering carbonation as a purely deleterious effect, the dissolution and ingress of CO_2_ into the pore solution lead to reduced pore-fluid pH and degradation of the cementitious material.^177^ In accelerated carbonation experiments, such as are commonly used to explore the degrees and mechanisms of carbonation, a lower resistance to carbonation is frequently recorded for AAMs compared to OPCs.^176,177^ However, such results are not necessarily applicable, as bicarbonate formation and the subsequent phase equilibrium alterations occurring at the high CO_2_ concentrations imposed in these tests can lead to higher pH reductions compared to natural carbonation.^72,79,196^ Hence, the more appropriate method to assess the carbonation-induced durability damage of GPs is to measure the natural carbonation depth of materials at a more realistic CO_2_ content.

Noteworthy is that most of the research analyzing the impacts of passive carbonation on AAMs has been performed on slag-based GPs.^72,249^ However, as noted above, Ca can play a complex role in carbonation processes, and how Ca content may affect the long-term carbonation resistance of a GP system remains unclear. On one hand, the addition of slag, as a high Ca content admixture, may improve the carbonation resistance of GP systems. For instance, Pasupathy et al.^245^ demonstrated the declined carbonation effects and improved durability of fly-ash-based systems modified with slag additives. Comparable results were obtained in the work conducted by Zhuguo and Sha.^249^ On the other hand, Badar et al.^250^ observed a higher degree of carbonation in GPs based on high Ca content fly ashes compared to those based on low Ca content fly ashes and proposed them as less suitable materials for CO_2_-rich environments. Similar results were obtained in the studies conducted by Song et al.^251^ and Bernal et al.,^173^ where a higher degree of carbonation was observed when slag was added to the GP systems. Such results are ascribed to the accelerated agglomeration of C(−A)–S–H species at higher Ca contents, which leads to quicker setting of GPs, hastening of geopolymerization, hindering the formation of N–A–S–H gels, and lower carbonation resistance of the system.^51,172−174^ In addition, in some studies, the reduced carbonation resistance is attributed to the formation of sodium and calcium carbonates and the subsequent formation of bicarbonates, with a reduction in pore fluid pH.^72,196,249^

These observed differences might arise from neglecting the effects of the activator concentration. It is demonstrated that at high Ca contents, increasing the NaOH concentration leads to the refinement of pore structure, lower permeability, declined depth of chloride penetration (decreased degree of efflorescence), and declined depth of CO_2_ diffusion (increased carbonation resistance).^51,174^ Note that using alkali contents above a certain value results in an increased degree of carbonation, and thus optimalization should be taken into account.^229^ Neglecting the role of particle fineness, curing temperature, and water content on carbonation resistance of GP systems may be another reason behind the contrary results reported in the literature.^174,249^ Consequently, further studies are required to explore how Ca/M (M: Na, K) contents of GPs affect the degree of carbonation, the properties of the cured GP, and the way each system interacts with CO_2_.

However, instead of being purely a deleterious effect, the carbonation of GPs could also lead to improvement of GP properties in certain applications. The solid carbonates precipitated during the carbonation process can act as pore filling materials that improve the compactness of the matrix and reduce the permeability (and thus the diffusion depth of CO_2_), strengthening the binder against further CO_2_ ingress.^114^ Pouhet and Cyr^72^ observed a rapid increase in compressive strength of metakaolin-based GPs experiencing accelerated degrees of carbonation, which was accompanied by a sharp decrease in pH values. This phenomenon was attributed to the OH^–^ bound to the structure of the material (more probably to silicon), which subsequently increased the mechanical strength of GPs. They also reported no harmful effects of carbonation on their cured GP after 365 days. To fully make use of these effects, Haq et al.^252^ even proposed the addition of sodium bicarbonate to the precursor, which would lead to the release of CO_2_ inside the GP during curing at temperatures between 150 and 200 °C.

However, when considering the beneficial effects of carbonate precipitation during CO_2_ exposure, selected studies have shown that during long-term exposure of Ca-rich sealant materials to CO_2_, once all available Ca from Ca(OH)_2_ and C(−A)–S–H gel has been consumed, a further decrease in pH can lead to subsequent dissolution of these carbonates. This results in increases in porosity and permeability (i.e., a reduction in resistance against carbonation) and deterioration of mechanical strength.^5,73^ Such deleterious effects in the long term thus need to be understood and prevented.

#### 3.4.3 Corrosion

Corrosion is a significant and widespread issue that can compromise the durability of systems containing steel components embedded within cement-based materials, such as OPC. The corrosion-induced challenges are expected to be even more severe in the highly corrosive environments encountered during CCS operations.^68,253,254^ GP systems have demonstrated promising properties that can help mitigate the adverse impacts of corrosion when employed either as the principal insulation material or as a coating material to provide protection for OPC-based cement and concrete.^79,255−259^ This section briefly presents the primary mechanisms of corrosion of steel embedded within isolation materials, the corrosion resistance of the GP systems, and the principal anticorrosion measures outlined in the literature.

##### 3.4.3.1 Main Mechanisms of Corrosion

In the process of steel corrosion, iron and other metallic components present in the steel interact with an oxygen source through a redox process, which poses a significant threat to the structural stability of the steel infrastructure. The corrosive activity can be further enhanced in CO_2_ wellbores, primarily due to the high temperatures and saline conditions prevailing in such environments.^72,79,254,260^ The resultant corrosion can potentially endanger the structural integrity of both the steel and the cementitious material used to embed the steel.

When steel is embedded in OPC-based cements/concretes, it is initially protected against corrosion by a thin oxide layer formed on its surface due to high alkalinity of the surrounding medium (with a pH ranging from 12.5 to 13.5).^245,261,262^ Although GP materials possess higher initial pH values than OPC, as well as relatively low permeability, they are still susceptible to chemical attack.^255−257,259,263^ This vulnerability can result in corrosion of the embedded steel in two stages. During the initiation stage, aggressive agents penetrate the cover zone and reach the steel surface, where they lead to the removal of the passivating oxide layer. Once this layer is removed, the propagation stage is initiated during which corrosion proceeds until eventually the steel component fails.^79,261^ The process of corrosion can cause a significant increase in the solid volume of the steel, leading to the generation of tensile stresses and fracturing of the cement seal surrounding the wellbore.^79,256,258,260,263,264^

The chemical mechanism underlying steel corrosion in the context of CCS operations involves two distinct processes. Chlorides instigate direct corrosion pitting by attacking the steel surface. In contrast, CO_2_ affects corrosion through its interaction with cementitious materials. The CO_2_ reacts with the free lime or alkali content present in these materials, resulting in carbonation and a consequent decrease in the pH of the system. This general pH reduction, in turn, leads to the dissolution of the passivating layer that protects the steel from further corrosion.^72,77,246,254,255,265,266^ Note that carbonation requires the presence of water and cannot proceed under completely dry conditions.^72,79,226,246,267^ Therefore, optimizing the properties of the sealant is crucial to minimize the potential for wellbore corrosion, as the optimized precursor materials, mix water content, hardener composition, and curing conditions ensure minimal porosity and permeability, thereby inhibiting the penetration of CO_2_ and chloride ions.^79,255,256,258,263^ Noteworthy is that, as curing at elevated temperatures can accelerate subsequent carbonation reactions, expected downhole (i.e., curing) temperatures need to be taken into account during mix design.^72,114,174,195,253,256,261,268,269^

In addition to CO_2_ and Cl^–^, sulfate dissolved in the pore brine can also contribute to steel corrosion through its deleterious impact on sealant integrity. Sulfate can react with calcium hydroxide (Ca(OH)_2_) to form calcium sulfate dihydrate (CaSO_4_·2H_2_O), thereby reducing the pH of the system in a similar way to CO_2_. Furthermore, sulfate can react with silica and alumina gels to form products that have no bonding strength.^258,263,265^ Note that these corrosion-enhancing mechanisms will augment each other in CCS applications, resulting in even more potential for corrosion.^254,261,263^

##### 3.4.3.2 Corrosion Resistance of GP Systems

Due to their higher alkalinity of GP systems and their position in the Pourbaix diagram, GPs may more durably passivate the embedded steel compared to OPC.^79,255−259^ However, the passivating capacity of GP systems may be influenced by factors such as the reactivity and composition of the precursor; the type, concentration, and molar ratio of the activator; and the curing conditions.^68,79,258,262−264^ The presence of Si and Al species in the pore fluids of GP systems can provide additional protection to depassivated steel and improve the corrosion resistance provided by GPs. Therefore, the Si/Al ratio of the system can influence the corrosion resistance of GPs.^68,258,270^ The higher alkalinity of GPs compared to OPC can also raise the minimum threshold of free chloride required for the onset of corrosion.^257^ Finally, the higher solubility of sodium bicarbonates and carbonates, compared to calcium carbonates, leads to the formation of a more effective pH buffer in the Na-containing pore solution of carbonated GPs, compared to the Ca-containing pore solution of carbonated OPC-based systems.^258^

In addition, the coexistence of N–A–S–H and C(−A)–S–H gels in GP materials leads to lower permeabilities compared to OPC, which translates to reduced penetration of corrosive agents.^68,256,257,265^ When considering Cl^–^, such penetration is further limited by the higher chloride binding capacity of N–A–S–H gel relative to C(−A)–S–H gel.^257,269,271,272^ Moreover, when Mg-containing additives are included, the formation of hydrotalcites, which possess a large surface area and high ion adsorption capacity, may further reduce Cl^–^ penetration and thus help inhibit chloride-induced corrosion.^255,256,259,264,269^

Despite the promising results reported in the literature, there are some contrasting findings where identical^262,272−275^ or even higher^253,263,265^ degrees of chloride attack or carbonation have been observed in GP materials compared to OPC. For example, Pasupathy et al.^263^ demonstrated that fly-ash-based GP concrete exposed to a saline environment for six years experienced lower resistance to carbonation, higher chloride penetration, and higher sulfate ingress compared to OPC. However, it was noted that these results may be specific to the particular mix design used and should not necessarily be generalized to all GP systems. In other words, suboptimal formulation with regards to chemical durability was likely responsible for the observed results.^256,262,263^

##### 3.4.3.3 Palliative Measures for Corrosion Protection

Apart from optimizing the mix design to provide a sealant with low permeability to corrosive agents, there are several palliative methods for corrosion protection. These methods include the use of inhibitors, protective coatings, cathodic protection, as well as the use of stainless steel or galvanized reinforcement.^79,276,277^

Utilizing stainless steels, which possess high corrosion resistance but at increased cost, and employing cathodic protection have been demonstrated to be the most effective methods for ensuring the durability of both the steel and the isolation system in aggressive environments. Galvanization of steel provides a cheaper alternative, with positive impacts on corrosion protection when compared to other preventative techniques, though the passivating layer formed during galvanization is more sensitive to environmental pH (and benefits from the presence of Ca^2+^).^79,278^ Furthermore, in GPs, the large concentration of free alkalis in the pore solution may diminish the durability of the zinc-based passivation layer.^257^

Corrosion inhibitors are also a viable and cost-effective option for extending both the initiation time (by increasing the threshold value of chloride or decreasing the penetration depth of chloride) and the propagation time (by decreasing the overall rate of corrosion).^79,261,264,276,279^ These materials can be classified based on their inhibition mechanism, as anodic (such as calcium nitrate, sodium nitrate, sodium benzoate, etc.), acting on the dissolution of steel, cathodic (such as sodium hydroxide, sodium carbonate, phosphates, silicates, etc.), acting on the reaction of oxygen on the surface of steel, or mixed inhibitors (such as materials with hydrophobic groups coupled with polar groups, organic polymers, etc.), acting through adsorption on the steel surface and creating a protective film.^79,280,281^

Finally, the addition of fiber reinforcement is another viable approach to enhance the durability of cementitious systems against chemical attack, particularly after (micro)cracking, as such fibers may stitch microcracks together, minimizing the volume of continuous voids and thus leading to a reduced loss of seal integrity when cracking does occur.^33,54,56,272,276,277^

In addition to their promising features as the main isolation materials, GPs have also demonstrated desirable characteristics when they are used as coating materials to protect OPC against corrosion. For instance, Zhang et al.^264^ studied the coating potential of a GP system (synthesized from 90 wt % metakaolin and 10 wt % GGBFS) and reported that the compact interface between the GP and cement, resulting from a significantly smaller pore size of GPs (94% pores < 20 nm) compared to OPC (73.7% pores > 50 nm), hindered the penetration of chloride.^255,257,258,263,276^

## 4 GPs as Wellbore Sealants

Although GPs show great potential as zonal isolation materials in laboratory-scale assessments, their field-scale application in realistic downhole conditions has yet to be known, and further research is needed to address the remaining uncertainties. Future research should focus on accurate assessment of the pore structure and chemical and mechanical durability of GPs when exposed to the high CO_2_ concentrations, saline water, and high stresses encountered during CCS operations.

### 4.1 Controlling GP Matrix Porosity and Permeability

Porosity and permeability are key parameters governing the durability of GPs as wellbore sealants, with lower porosities yielding higher compressive strength, reduced thermal expansion, and higher durability. Usually, lower porosities also give lower permeabilities and thus a system that can better resist chloride attack and CO_2_ penetration.^52,135^ The key factors affecting the porosity (and permeability) of a GP are as follows:

- Water to solid ratio: Increasing the water content in a GP system can cause an increase in porosity and permeability due to the enhanced mobility of ions, destabilization of Al tetrahedra, and increased susceptibility of the system to fracturing (as discussed in Section 3.2).^127^ Conversely, a decrease in the water-to-solid ratio, for a given composition, can significantly decrease the porosity and permeability of the GP, leading to improved microstructures.^50,63^ However, a decrease in water content beyond a certain point can result in drying shrinkage and the development of microcracks, ultimately resulting in increased permeability of the GP to aggressive chemical agents and susceptibility to corrosion-induced damage.^103,236^ It is also important to note that GP activation and polymerization are sensitive to water content, which may lead to increased porosity at suboptimal water contents.^46−49^
- Alkali content: Recent studies clearly show the reduced porosity of GPs at increased reaction rates obtained through increasing the alkali content. However, as noted above, excess alkalinity can impact the charge balance of GPs and lead to long-term leaching and lower system durability.^50^ Furthermore, using mixed hardeners containing both waterglass and alkali hydroxides leads to the formation of high silicon content gels, resulting in increased compactness and reduced porosity and permeability of GP materials compared to those activated solely with alkali hydroxides.^75,88,138,139^
- Curing conditions: GP systems synthesized at low curing temperature, due to slow polycondensation and polymerization, benefit from improved qualities in terms of porosity and toughness.^44^ Increasing the curing temperatures, up to an optimum value, results in partial crystallization, yielding lower porosity and pore interconnectivity.^75,79,126^ However, prolonged curing at high temperatures can result in increased heterogeneity of the pore structure, increased pore radii, increased evaporation of water, distorted reaction, the development of microcracks, and mechanical failure.^44,63,215,227,236^
- Reactivity of precursors: The porosity and (CO_2_) permeability of a GP are directly linked to the reactivity of its precursors, which in turn depends on surface area (i.e., grain size and shape) and degree of crystallinity of SiO_2_ and Al_2_O_3_.^51^ Precursor particle shape and fineness, through their control on surface area, substantially affect the reaction rate but also impact water demand, strength development, and homogeneity of GPs. In fact, more spherical particles, such as fly ashes, have the least possible surface area per unit volume and thus have a low water demand, while using precursors with irregular-shaped or plate-shaped particles, such as clays and metakaolin, leads to increased water demand and higher porosity of the GP system.^51,52,204^ Enhancing particle fineness will similarly lead to higher reactivity of the precursor.^37,43,78^ As water demand and precursor reactivity are impacted by not only mean particle size but also particle size distribution and particle shape, the water demand of a mix needs to be assessed accurately through fully controlled experiments.^63^
- Additives: Numerous studies have recently shown the applicability of nanomaterials in reducing GP porosity and permeability and thus enhancing their durability and mechanical strength while limiting shrinkage.^52,93,146,164^ Furthermore, fibers, by protecting air bubbles and improving the bonding of particles, can increase GP porosity while also reducing the risks of crack development and enhancing the ductility of cured GP systems, by improving their capacity to dissipate strain.^33,56,91,272^ The implementation of palliative techniques for corrosion inhibition, as discussed in Section 3.4.3, can also help to ensure that the system maintains a low level of porosity and permeability over an extended period of time.^261,276,279^

### 4.2 Durability of GP Exposed to Brine and CO_2_

A limited number of studies have been performed to investigate the behavior of GP systems cured in brine solutions and/or CO_2_-rich conditions. As shown in Table 3, with some exceptions,^33,68^ the general agreement is that GPs cured in brine solutions show better mechanical durability compared to those cured in water and that the strength reduction of GPs in both water and brine is lower than that of OPC. As discussed in Section 3.4, the reduced alkali leaching of GPs, especially at higher curing brine salinity, is given as the major reason for these observations.^2,7,8,33,67,68,282^

**Table 3:** Summary of Published Studies Addressing the Mechanical Properties of GP Systems Exposed to Conditions Representative of Those Encountered During CCS Operations

Author(s)	Precursor	Alkali metal	Alkali concentration (wt %, M)	Curing/exposed temperature	Other conditions	Main achievement(s)
Nasvi et al.^32^	FFA^a^	Na	Combination of 10 M NaOH and Na_2_SiO_3_	50 °C	Samples were cured at CO_2_ chambers (3 MPa) up to 6 months.	No significant changes in microstructure and compressive strengths were observed.
						Na_2_SiO_3_/NaOH ratio = 2.5
Nasvi et al.^2^	FFA	Na	Combination of 10 M NaOH and Na_2_SiO_3_	50 °C	Samples were cured at solutions with NaCl contents of 5 and 15% for 24 h.	Due to reduced alkali leaching, the strength reduction in GP samples was almost half of that of OPC.
Giasuddin et al.^7^	FFA	Na	Combination of 8 M NaOH and Na_2_SiO_3_	Ambient temperature	-After hardening at ambient temperature, samples were immersed in the solutions with NaCl contents of 0, 8, and 15% for 28 days.	Due to reduced alkali leaching, GPs cured at saline water benefited from higher compressive strengths compared to the samples cured at fresh water.
	GGBFS				-Na_2_SiO_3_/NaOH ratio = 2.5	OPC samples cured at saline water exhibited a sharp decline in compressive strength.
Barlet-Gouedard et al.^284^	Metakaolin	Na	Combinations of NaOH and Na_2_SiO_3_	90 °C	GPs were exposed to CO_2_-saturated water and wet scCO_2_ for 15 days and under curing temperature and pressure of 90 °C and 28 MPa, respectively.	Excellent mechanical properties were obtained after wet scCO_2_/CO_2_-saturated water exposure, and no significant degradation was observed in GP microstructures.
Prusty and Pradhan^68^	FFA	Na	Combination of 12 M NaOH and Na_2_SiO_3_	Ambient temperature, 80 °C	GPs were prepared through mixing FFA and GGBFS with 0 and 3.5 wt % of NaCl.	The initial strength of FFA-GGBFS-based GPs was higher than that of FFA-based GPs.
	GGBFS				-GPs were cured at ambient temperature for 48 h followed by curing at 80 °C for another 48 h.	A 7.86% decrease in the compressive strength of FA-based GPs and 30.24% decline in that of FA-GGBFS-based specimens were observed. Strength decreases were attributed to the impacts of salt crystallization and precipitation.
					-Na_2_SiO_3_/NaOH ratio = 1.5	
Ren et al.^33^	Metakaolin, wollastonite, short basalt fiber, and tremolite	Na	Combination of NaOH and Na_2_SiO_3_	28 °C	GPs were exposed to a range of NaCl content of 0–20 wt % at 28 °C and for 3, 7, 28, and 90 days.	Decrease in compressive strength after exposure to NaCl was augmented with the extended exposure period. The strength loss was directly linked to microcracks developed in the specimens structure.
Khalifeh et al.^67^	Aplite rock, GGBFS, and microsilica	K	Combination of 8 M NaOH and Na_2_SiO_3_	Ambient temperature, 100 °C	-GPs were cured at ambient temperature for 1 week.	Permeability of GPs was low enough after exposure to brine and their compressive strength started to rise after six-month exposure.
					-Then samples were exposed to an NaCl content of 2.45% (and oil, and H2S) at 100 °C and for up to 12 months.	The samples exposed to H_2_S experienced serious degradation.
					-Na_2_SiO_3_/NaOH ratio = 1	
Ridha et al.^239^	CFA^b^	Na	Combination of 8 M NaOH and Na_2_SiO_3_	60 °C	-GPs were exposed to wet CO_2_ at 17.23 MPa/60 °C and 24.13 MPa/130 °C for 72 and 24 h, respectively.	After CO_2_ exposure, GP samples showed higher compressive strengths compared to OPC. Also, accelerated carbonation at higher temperatures had a negative impact on the strength of CO_2_ exposed specimens.
				–130 °C	Na_2_SiO_3_/NaOH ratio = 2.27	

a Class-F fly ash.

b Class-C fly ash.

Moreover, most research revealed that compared to OPC systems GPs exposed to CO_2_-rich conditions benefit from lower CO_2_ permeability and an appropriate degree of mechanical durability. For instance, using triaxial tests conducted at 26 °C with CO_2_ injection pressures in a range of 3–13 MPa, Nasvi et al.^232^ showed that the CO_2_ permeability of selected fly-ash-based GPs was in a range between 0.002 and 0.06 μD. In another work, in an attempt to evaluate the durability of a set of fly-ash-based GP systems with different concentrations of alkali activated slag, Nasvi et al.^283^ showed that the CO_2_ permeability of GP systems (0.0005–0.002 μD) was at least 2–3 orders lower than that of selected OPC (0.12–2.6 μD), and even the CO_2_ permeability of the GPs containing 15% slag was 1000 times lower than that of the OPC. In further work, Nasvi et al.^44^ found sharp increase in the CO_2_ permeability of GPs at elevated curing temperatures, with increment rates between 200 and 1000%; however, even the highest obtained CO_2_ permeability (0.04 μD) was well below that of selected OPC and the limits recommended by the American Petroleum Institute (API) for typical wellbore isolation systems (0.2–200 μD).^232^ In addition, carbonation appears to have a much smaller impact on GP properties compared to OPC. For example, Nasvi et al.^32^ reported a very minor 2% reduction in the compressive strength of selected fly-ash-based GPs exposed to a CO_2_-rich environment at 3 MPa for up to 6 months, while SEM analysis showed almost no noticeable change in the microstructure of the exposed samples. Likewise, Barlet-Gouedard et al.,^284^ who exposed selected metakaolin-based GPs to CO_2_-saturated water and wet scCO_2_ for 15 days at representative in situ conditions for CCS wellbores (90 °C and 28 MPa), reported outstanding mechanical properties and no significant microstructure degradation for cured samples.

## 5 Challenges, Research Gaps, and Perspectives

### 5.1 Challenges

The practical implementation of GP systems in downhole operations faces several challenges that require further fundamental research. A significant challenge in the realm of GP materials is the achievement of precise control over their properties during both the slurry phase and the subsequent hardened state. Specifically, a systematic optimization approach is essential to determine the optimum molar ratios of starting materials (Si/Al, Si/(Na+K), Si/Ca, etc.) and water content required to achieve the best performance of GP-based sealants under different curing conditions. In addition, further challenges identified include the long-term durability of GPs (especially under harsh environments such as prolonged high/cyclical temperature, high pressure, and corrosive conditions), achieving consistent and reproducible GP properties, from precursors with (naturally) varying properties, and a complete understanding of corrosion mechanisms and long-term performance of the steel–GP system under downhole conditions.

### 5.2 Research Gaps

The role of water in the geopolymerization reaction and the effect of water content on the properties of GPs as well as lack of knowledge about the impact of factors such as GP matrix chemical composition, curing conditions and methods, and exposure to aggressive conditions on the aging of GP seals are the main research gaps identified here. Significant research gaps also exist concerning the effects of thermal cycling induced stresses on GP systems and the influence of chemomechanical processes, such as drying-induced salt precipitation as well as hydrophysical processes, particularly the impacts of wettability alteration on the long-term rate of chemical attack. Finally, additional research is required to address the effects of impurities in the raw materials on the properties and durability of GPs and the fundamental mechanisms of geopolymerization and to develop predictive models for GP formation and subsequent development.

### 5.3 Perspectives

GPs have the potential to be used in a variety of applications beyond construction, including as sealants in wellbore construction or plugging and abandonment. In these applications, GPs benefit from their ability to be tailored for the specific properties required to endure under the conditions to which they will be exposed by adjusting factors such as precursor composition, curing conditions, and chemical admixtures. Their chemical (e.g., low Ca content) and physical properties (e.g., ductility and low matrix permeability) are suitable for well cementing applications when the slurry is properly designed. Finally, utilization of low CO_2_ intense technology for replacement of oil well cement can help the oil and gas industry to achieve its net-zero emission target.

## 6 Conclusions

After reviewing the literature on geopolymer (GP) designs, with a focus on properties desirable for GPs used as wellbore sealants in carbon capture and storage (CCS) applications, it is concluded that properly formulated GPs can exhibit promising features such as high ductility, low shrinkage, and high resistance against carbonation. However, further investigation is required to optimize the selected GP systems. Such optimization of GP compositions should prioritize achieving a dense microstructure, with low (interconnected) porosity and thus low CO_2_ permeability, and sufficient chemical durability when exposed to CO_2_ in wet environments. Key parameters that can be optimized to obtain these desired properties include the water content; chemical composition and particle size distribution of the precursor; and chemical composition of the hardener, while taking into account the downhole (curing) temperature and salinity conditions.

- Excess water content can impede geopolymerization and polycondensation, increase cation mobility, and destabilize [AlO_4_]^−^ tetrahedra. This may result in inhibited hardening and increased rates of efflorescence and carbonation, ultimately leading to reduced chemical durability. The optimum water should strike a balance between required strength development and the desired workability and viscosity of the system. The optimum value appears to be below 35 wt % in most of the mix designs.
- The particle size distribution of its precursors strongly impacts the final microstructure of a GP, with finer particles resulting in increased reactivity, which in turn yields decreased porosity and permeability. However, increasing the fineness of the precursor particles may lead to a higher water demand, especially when milling (or similar techniques) results in irregular particle shapes.
- With regards to systems chemistry:
  a Generally, Si/Al ratios between 2 and 3 are considered optimal for achieving good mechanical properties and durability in most GP systems. However, some studies have shown that lower Si/Al ratios (between 1.5 and 2) may be more suitable for specific applications such as wellbore cementing. Optimum Si/Al ratios also depend on factors such as curing temperature, RH, particle size and reactivity of the precursor, and system alkalinity. Observed trends can be further complicated by the Ca content of the GP system.
  b The Si/M and Al/M ratios (where M:Na+K) also affect the mechanical durability and setting behavior of GPs by controlling the dissolution rate and densification of the microstructure. In an optimization process, the negative impacts of high alkali content on the potential for carbonation and efflorescence need to be balanced against the impediment of geopolymerization experienced at low alkali contents.
  c The Si/Ca ratio controls the formation of relatively dense C(−A)–S–H gels that fill voids and are commonly acknowledged to enhance mechanical strength. However, elevated Ca contents can also have deleterious impacts on the GP microstructure due to the provided extra nucleation cites and extremely reduced setting times. Furthermore, high Ca contents can make GP systems more sensitive to CO_2_ exposure and carbonation. The effect of Ca content on the rate of efflorescence is not fully understood and needs further research.
  d The Si/Mg and Si/Fe ratios can affect the strength development of GP systems. The filler effects of MgO can lead to increased strength, through the formation of Mg(OH)_2_ and the precipitation of expansive hydrotalcite-like phases that can reduce pore sizes during hardening. However, the presence of Mg impurities can also lead to the creation of microcracks and the weakening of Ca bonds in C(−A)–S–H gels. Excess MgO content might also result in the increased porosity of the system. In terms of Si/Fe ratio, improved mechanical properties are ascribed to filler effects of Fe_2_O_3_ species and the formation of iron-silicate, polysialate, and ferro-sialate (Fe(−Al)–S–H) phases. However, rapid precipitation of Fe (hydr)oxides during hardening, and the associated consumption of OH^–^, may lead to decelerated dissolution of the remaining precursor silicates, impeding further geopolymerization.
- The optimum curing temperature for most GP systems appears to be between 60 and 80 °C. Curing at low temperatures result in slow GP growth, leading to low porosity and permeability. On the other hand, curing at elevated temperatures, up to a system-dependent optimum value, can improve consolidation time and yield more rapid compressive strength development and reduced pore sizes, in turn leading to reduced efflorescence and improved carbonation resistance. Furthermore, it is shown that the optimum curing temperature of a GP can be affected through adapting parameters such as the Si/Al ratio, hardener chemistry, and precursor particle size distribution, meaning that GPs can be developed to operate optimally at higher temperatures. Such optimization is also required to address challenges to GP integrity caused by prolonged curing at high temperatures.

This literature review on the applicability of GP systems as wellbore sealants for CO_2_-rich environments shows that while GP technology offers many promising features for such applications thorough optimization of such systems is essential. While key parameters have been identified through which GP systems can be optimized, important questions remain regarding the impact of these parameters on the durability and mechanical properties of a GP under realistic CO_2_-rich conditions, which require further study.
